# TRAIL-Receptor 4 Modulates γδ T Cell-Cytotoxicity Toward Cancer Cells

**DOI:** 10.3389/fimmu.2019.02044

**Published:** 2019-08-28

**Authors:** Doaa Tawfik, Christopher Groth, Jan-Paul Gundlach, Matthias Peipp, Dieter Kabelitz, Thomas Becker, Hans-Heinrich Oberg, Anna Trauzold, Daniela Wesch

**Affiliations:** ^1^Institute for Experimental Cancer Research, Christian-Albrechts-University of Kiel, Kiel, Germany; ^2^Institute of Immunology, University Hospital Schleswig-Holstein, Christian-Albrechts University of Kiel, Kiel, Germany; ^3^Department of General Surgery, Visceral, Thoracic, Transplantation and Pediatric Surgery, UKSH, Campus Kiel, Kiel, Germany; ^4^Division of Stem Cell Transplantation and Immunotherapy, Department of Medicine II, UKSH, CAU Kiel, Kiel, Germany

**Keywords:** TRAIL-receptor 4, γδ T cell-cytotoxicity, pancreatic cancer, COX (cyclooxygenase), PGE2, granzyme B, TRAIL, bispecific Ab

## Abstract

Acquired immune evasion is one of the mechanisms that contributes to the dismal prognosis of cancer. Recently, we observed that different γδ T cell subsets as well as CD8^+^ αβ T cells infiltrate the pancreatic tissue. Interestingly, the abundance of γδ T cells was reported to have a positive prognostic impact on survival of cancer patients. Since γδ T cells utilize TNF-related apoptosis inducing ligand (TRAIL) for killing of tumor cells in addition to granzyme B and perforin, we investigated the role of the TRAIL-/TRAIL-R system in γδ T cell-cytotoxicity toward pancreatic ductal adenocarcinoma (PDAC) and other cancer cells. Coculture of the different cancer cells with γδ T cells resulted in a moderate lysis of tumor cells. The lysis of PDAC Colo357 cells was independent of TRAIL as it was not inhibited by the addition of neutralizing anti-TRAIL antibodies or TRAIL-R2-Fc fusion protein. In accordance, knockdown (KD) of death receptors TRAIL-R1 or TRAIL-R2 in Colo357 cells had no effect on γδ T cell-mediated cytotoxicity. However, KD of decoy receptor TRAIL-R4, which robustly enhanced TRAIL-induced apoptosis, interestingly, almost completely abolished the γδ T cell-mediated lysis of these tumor cells. This effect was associated with a reduced secretion of granzyme B by γδ T cells and enhanced PGE2 production as a result of increased expression level of synthetase cyclooxygenase (COX)-2 by TRAIL-R4-KD cells. In contrast, knockin of TRAIL-R4 decreased COX-2 expression. Importantly, reduced release of granzyme B by γδ T cells cocultured with TRAIL-R4-KD cells was partially reverted by bispecific antibody [HER2xCD3] and led in consequence to enhanced lysis of tumor cells. Likewise, inhibition of COX-1 and/or COX-2 partially enhanced γδ T cell-mediated lysis of TRAIL-R4-KD cells. The combination of bispecific antibody and COX-inhibitor completely restored the lysis of TRAIL-R4-KD cells by γδ T cells. In conclusion, we uncovered an unexpected novel role of TRAIL-R4 in tumor cells. In contrast to its known pro-tumoral, anti-apoptotic function, TRAIL-R4 augments the anti-tumoral cytotoxic activity of γδ T cells.

## Introduction

The prominent role of death ligands Tumor necrosis factor (TNF)-α, TNF-related apoptosis inducing ligand (TRAIL) and CD95-L in tumor biology is undoubted, yet not fully understood. Particularly, the TRAIL/TRAIL-R system remains still widely unexplored, due to its complexity. There are four plasma membrane expressed TRAIL receptors, TRAIL-R1-R4 and one soluble TRAIL receptor, Osteoprotegerin (1–6). In addition to binding of TRAIL, all plasma membrane receptors can interact with each other forming homo- and hetero-complexes and this can take place prior to and/or following TRAIL-binding. Two of the receptors, TRAIL-R1 and TRAIL-R2 carry the so-called death domain and are therefore able to induce cell death. This function of TRAIL-R1/-R2 is believed to be important for the immune surveillance of tumors. Accordingly, down regulation of TRAIL-R1 and/or TRAIL-R2 at the cell surface are characteristic features of cancer cells leading to tumor escape and acquired resistance of tumor cells toward TRAIL-induced apoptosis (7, 8). In addition, to inducing cell death, TRAIL-R1/-R2 are able to trigger several non-apoptotic signaling pathways which may increase tumor malignancy by influencing the tumor cells themselves as well as the tumor microenvironment via secreted inflammatory cytokines (1, 9–12). Two additional TRAIL receptors, TRAIL-R3 and TRAIL-R4, do not contain a functional death domain and therefore unable to induce cell death. Instead, these receptors negatively regulate TRAIL-induced apoptosis via binding of TRAIL and/or interaction with TRAIL-R1/-R2 (2–4, 13, 14). Of note, through their role as decoy receptors, TRAIL-R3, and TRAIL-R4 can act either autonomously at the cell or at a supracellular level thus influencing the sensitivity of other cells in the tumor microenvironment to TRAIL-induced apoptosis (15).

Importantly, TRAIL death receptors are frequently overexpressed in tumors but instead of being present at the plasma membrane they are mainly localized intracellularly, in the cytoplasm and in the nucleus. Emerging evidence suggests that these intracellular TRAIL receptors exert functions different from that of the plasma membrane expressed receptors and can influence or contribute to the malignant progression (7, 16–22). Accordingly, it has been shown for some tumor entities that high intracellular expression of mainly TRAIL-R2, also TRAIL-R1, correlates with poor patients' prognosis (7).

Only recently, specific functions of intracellular TRAIL receptors were uncovered. Thereof, nuclear TRAIL-R2 interacting with the microprocessor complex regulates the maturation of miRNA let-7 and thereby promotes tumor cell proliferation (19). In addition, cytoplasmic TRAIL-R1 and TRAIL-R2 have been shown to induce apoptosis in response to the unresolved unfolded protein response (23, 24).

In contrast to the plethora of data dealing with TRAIL-R1/-R2 functions in tumor cells, the function of TRAIL-R4 is largely unexplored and is almost exclusively deduced from overexpression studies. Unlike TRAIL-R3, which is only anchored in the plasma membrane and is therefore unable to induce intracellular signaling, TRAIL-R4 contains a complex intracellular part which is homologous to those of TRAIL-R1 and TRAIL-R2 but its death domain is truncated and therefore non-functional (2, 4). Consistently, several reports showed that beside the ability to inhibit TRAIL-mediated apoptosis TRAIL-R4 can induce non-apoptotic signaling pathways like NF-κB and AKT, which might contribute to its pro-tumoral function (2, 25). This might explain the reported overexpression of TRAIL-R4 in tumors, which in some cases could be correlated to a more malignant phenotype and poor patients' prognosis (17, 26, 27). On the other hand, some tumors down regulate the expression of TRAIL-R4, suggesting a context-dependent and/or cancer specific function of this receptor (22, 28, 29).

Tumors and the tumor microenvironment contain a network of immunoregulatory and immunosuppressive mediators which influence effector functions of tumor-infiltrating cells including γδ T cells. γδ T cells play a major role in immune surveillance by recognizing overproduced metabolites and stress-induced surface molecules on cancer cells. Aside from the classical cytotoxic mediators (e.g., granzymes), death ligands TNF, CD95, and TRAIL are used by the different γδ T cell-subsets to lyse a broad range of tumor cells (30). γδ T cells represent a numerically small population of T cells in the peripheral blood with a predominance of Vγ9Vδ2 T cell receptor (TCR)-expressing T cells. Vγ9Vδ2 T cells recognize pyrophosphates of the isoprenoid pathway of prokaryotes or dysregulated mevalonate pathway of tumor cells in a butyrophilin 3A (BTN3A)-dependent manner (31, 32). The Vδ1 γδ T cells are rare in the peripheral blood of healthy donors but are frequently found in cancer patients (30, 33–35). Vδ1 T cells express one of various Vγ chains (Vγ 2, 3, 4, 5, or 8) and recognize microbial- and self-lipids bound to CD1d molecules or stress-induced MHC class-I related chain A (MICA), that are frequently expressed on tumor cells including pancreatic ductal adenocarcinomas cells (PDAC) (36–38). Both γδ T cell-subsets highly express NKG2D, the activating receptor for MICA and MICB and lyse different tumor cells in a NKG2D-dependent manner (39). Shedding of NKG2D ligands from PDAC cells is reported as one of the tumor escape mechanism (36). Beside the interaction of NKG2D with MICA or MICB, the interaction with other adhesion- and costimulatory molecules such as CD54, CD80/86, CD154, and their corresponding ligands CD11a/CD18, CD28, and CD40 expressed on γδ T cells are conducive for triggering their effector function (40, 41). Both γδ T cell-subsets recognize their antigens in a HLA-independent manner and infiltrate tumors which is the underlying principle of γδ T cell-based immunotherapies (42–45). The abundance of intratumoral γδ T cells was identified as one of the most important prognostic factor associated with a positive outcome in a recent meta-analysis (46). While they exert pronounced antitumor efficacy and therefore are potential candidates for immunotherapy, Vδ2- as well as Vδ1 γδ T cells can also be inhibited by immunosuppressive cells and mediators such as cytokines in the tumor-microenvironment as well as by different inherent tumor escape mechanisms (47). Recently, we observed that the majority of PDAC cells are highly resistant to Vδ2 T cell-mediated lysis, which can be explained by different mechanisms [(48) and unpublished observations]. In this context, we demonstrated that the coculture of PDAC Colo357 and other tumor cells such as breast cancer, MDA-MB-231, and cervical cancer HeLa cell lines with Vδ2 T cells increased the prostaglandin (PG) synthetase cyclooxygenase (COX)-2 expression in tumor cells and thereby the release of PGE2, which in turn significantly inhibited Vδ2 T cell-mediated cytotoxicity (48). Similar to Vδ2 T cells, Vδ1 T cells infiltrate tumors and their activity could be regulated by different mechanisms preventing their effective anti-tumor response. Since γδ T cells may use TRAIL to kill tumor cells, we investigated the role of TRAIL/TRAIL-R system with a focus of TRAIL-R4 on the sensitivity of tumor cells toward γδ T cell-induced cytotoxicity.

## Materials and Methods

### Establishment of γδ T Cell Lines

γδ T cell lines were established from peripheral blood mononuclear cells (PBMC) of healthy adult donors (#1 and #2 in Figures 2A, 3; additional donors in Figures 2B, 7, 8) and cancer patients (#3 in Figures 2, 3; additional donors in Figures 2B, 7, 8). PBMC were isolated from leukocyte concentrates obtained from healthy blood donors and provided by the Department of Transfusion Medicine of the University Hospital Schleswig-Holstein (UKSH) in Kiel, Germany. In addition, heparinized blood from cancer patients was obtained from the Department of General Surgery of the municipal hospital. In accordance with the Declaration of Helsinki, written informed consent was obtained from all donors, and the research was approved by the relevant institutional review boards (ethic committee of the Medical Faculty of the CAU Kiel, code number: D445/18).

PBMC were isolated by Ficoll-Hypaque (Biochrom, Berlin, Germany) density gradient centrifugation and cultured in RPMI 1640 supplemented with 2 mM L-glutamine, 25 mM HEPES, 100 U/mL penicillin, 100 μg/mL streptomycin, 10% fetal bovine serum (FBS) [complete medium]. To expand Vδ1-expressing γδ T cells, 24-well plates were coated with 100 μL of 0.5 μg/mL anti-Vδ1 TCR monoclonal antibody (mAb) clone R9.12 (Beckman Coulter, Krefeld, Germany) overnight at 4°C. After washing the wells, 10^6^ PBMC/well were cultured with a final concentration of 1 μg/mL anti-CD28 mAb clone CD28.2 (Biolegend, Fell, Germany) and 50 U/mL rIL-2 (Novartis, Basel, CH) for 14 to 21 days. Since resting, initially stimulated Vδ1 γδ T cells produced only low amounts of IL-2, 50 U/mL rIL-2 were added every other day. After 2–3 weeks, Vδ1-expressing γδ T cell lines had a purity >40–60% and were labeled with anti-TCRαβ mAb clone IP26 (Biolegend) and subjected to magnetic separation in order to deplete remaining αβ T cells.

Additional large-scale expansion of Vδ1 T cells (that continued to coexpress various Vγ-chains) was performed in rIL-2-supplemented medium with repetitive restimulation using 0.5 μg/mL phytohaemagglutinin (Thermo Fisher Scientific, Langenselbold, Germany) and irradiated PBMC (20 × 10^6^ cells) and/or EBV-transformed B cell lines (2 × 10^6^ cells) as feeder cells for 20 × 10^6^ cells Vδ1 T cells. Dead feeder cells were removed 3–4 days after restimulation by Ficoll-Hypaque density gradients. Purity of Vδ1 γδ T cells was >98% as analyzed by flow cytometry.

### Tumor Cell Lines, Establishment of Clones, and Cell Culture Conditions

Human PDAC cell lines PancTuI and Colo357 as well as breast cancer cell line MDA-MB-231 were cultured in RPMI 1640 medium with 2 mM glutamine, 1 mM sodium pyruvate (all from Gibco, Darmstadt, Germany) and 10% FBS (PAN Biotech, Aidenbach, Germany) under regular conditions (5% CO_2_, humidified, 37°C). In addition, cervical cancer cell line, HeLa cells, with TRAIL-R4 overexpression and the appropriate control cells were established and gratefully provided by Prof. Olivier Micheau (INSERM, Dijon, France). HeLa cells were cultured in DMEM with 4.5% D-Glucose with 10% FBS (PAN Biotech), 2 mM Glutamine and 1 mM sodium pyruvate and 2.5 μg/mL puromycin for selection under regular conditions.

For stable knockdown of TRAIL-R4, cells were transduced with the GIPZ Lentiviral Human TNFRSF10D shRNA or with the non-silencing control [cloneIDs: V2LHS_16774 (led to the establishment of clone (a), V2LHS_16773, V3LHS_344449 (led to the establishment of clone (b), V3LHS_344451; Dharmacon, GE Healthcare, Lafayette, CO, USA] and selected with puromycin (Colo357/MDA-MB-231 cells with 2 μg/mL and PancTuI cells with 1 μg/mL). Additionally, the same method was used to knockdown TRAIL-R1 (cloneID: V3LHS_383714) and TRAIL-R2 (cloneID: V2LHS_16711). To inhibit cell signaling pathways, cells were seeded in 6-wells (5 × 10^5^/well and 24 h later treated for 8 h with 2 μM Insolution™ MG-132 proteasome inhibitor (#474791, EMD Millipore Corp., USA), 10 μM MEK inhibitor U0126 (Promega, Madison, USA) or 1 μM MK-2206 2HCL AKT-1, -2, -3 inhibitor (#S1078, Selleckchem, USA).

Absence of mycoplasma was routinely confirmed by RT-PCR (Venor® GEM classic, Minerva Biolabs GmbH, Germany).

### RNA Interference

8.5 × 10^6^ HeLa cells were seeded in 15 cm Petri dish, and incubated for 24 h under normal conditions. The cells were transfected with 10 μM TRAIL-R4 siRNA or non-targeting control pool, on-Targetplus, human siRNA, smartpool (TNFRSF10D; L-008092-01-0005, non-targeting; D-001810-10-20, both from Dharmacon/Horizon Discovery, USA) using Lipofectamine® RNAiMAX reagent (ThermoFisher Scientific, USA) according to manufacturer's Protocol.

### Real-Time Polymerase Chain Reaction

Cells were homogenized with QIAshredder (QUIAGEN, Hilden, Germany) and total RNA was isolated with a RNeasy Mini Kit (QUIAGEN). cDNA synthesis was performed using Maxima first strand cDNA synthesis kit (K1671, Thermo Fisher Scientific, USA). The expression of TRAIL-R4 was determined by qPCR using TaqMan assays (Thermo Fisher Scientific, USA) and a 7900HT Fast qPCR system (Thermo Fisher Scientific, USA). The expression levels were calculated relative to the expression of the housekeeping gene TATA-binding protein (TBP) by ΔΔCT method. Primers were purchased from Thermo Fisher Scientific (TBP (Hs00427620_mL) and TRAIL-R4 (Hs00388742_mL).

### Flow Cytometry

For the analysis of the purity of the Vδ1 γδ T cells, cells were stained with the following mAb: anti-CD3 (clone SK7, BD Biosciences, Heidelberg, Germany), anti-TCRγδ (clone 11F2, Miltenyi Biotech, Bergisch Gladbach, Germany), anti-TCRαβ (clone IP26, Biolegend), anti-TCRVδ2 (clone Immu389, Beckman Coulter), anti-TCRVδ1 (clone TS8.2, Thermo Fisher Scientific), anti-TCRVγ9 [clone 7A5, (49)], anti-TCRVγ2, -3 or -4 [clone 23D12, (50)], anti-TCRVγ8 [clone R4.5.1 (51)] or corresponding isotype controls (BD Biosciences or Biolegend).

For surface staining of adhesion-and costimulatory molecules and TNF-receptor family members, 2 × 10^5^ cells were washed and stained with mAb as follows: anti-CD44 (clone HCAM, BD Biosciences), anti-CD54 (clone 84H10, Beckman Coulter), anti-CD80 (clone L307.4, BD Biosciences), anti-CD86 (clone 37301, R&D Systems, Wiesbaden, Germany), anti-CD154 (clone 24–31, Ancell), anti-MICA (clone 159227, R&D Systems) and anti-MICB (clone 236511, R&D Systems) or appropriate isotype controls for 25 min. After two washing steps, cells were measured by flow cytometry.

For intracellular staining, 2 × 10^5^ tumor cells were treated (or not) with 1 μg/mL bispecific antibody (bsAb) [HER2xCD3] overnight, washed, permeabilized and fixed with Cytofix/Cytoperm kit (BD Biosciences) and stained with anti-COX-1-FITC/anti- COX-2-PE mAb (clone AS70/AS67, BD Biosciences) or with unconjugated COX-1 (clone Cox111, Thermo Fisher Scientific) followed by second-step with PE labeled goat-anti-mouse (Thermos Fisher Scientific) or the appropriate isotype controls following the procedures outlined by the manufacturer. After washing, all samples were analyzed on a LRS Fortessa flow cytometer (BD Biosciences) using DIVA 8.0 software.

For TRAIL-R cell surface expression analysis, one million cells were directly stained with APC-labeled mouse mAb (all from R&D Systems, biotechne) as follows: IgG1 isotype control (clone #11711) or anti-TRAIL-R1/TNFRSF10A (clone #69036), anti-TRAIL-R3/TNFRSF10C (clone #90906), and TRAIL-R4/TNFSF10D (clone #104918) as well as with IgG2B isotype control (clone #71908) or anti-TRAIL-R2/TNFRSF10B (clone #71908) and analyzed on a FACS Calibur using Cell-Quest Software.

### Western Blot Analysis

Cells were lysed in RIPA buffer supplemented with Complete Protease Inhibitor Cocktail and PhosphoStop (both from Roche, Mannheim, Germany) and Western blot analyses were performed as described previously (10). Primary antibodies were purchased from: Cell Signaling, Frankfurt, Germany [anti-ERK1/2 (9102), anti-phospho-ERK1/2 (9106), anti-TRAIL-R4 (8049), anti-TRAIL-R2 (3696), anti-mouse-IgG-HRP (7076), anti-rabbit-IgG-HRP (7074)]; EMD Millipore Corp., USA [TRAIL-R1 (AB16955)], BD Biosciences, USA [anti-COX-2 (610203)], TRAIL-R3 (DcR1, im-245-1, Imgenex, San Diego, USA), HSP90α/β (Santa Cruz, sc-7947), and from Sigma-Aldrich [anti-β-actin (A5441)].

### Real Time Cell Analyzer

Cytotoxicity against adherent cancer cells was measured by a Real Time Cell Analyzer (RTCA, X-Celligence, ACEA, San Diego, CA, USA) in triplicates as described elsewhere (34, 44, 52, 53). To monitor the impedance of the cells via electronic sensors on the bottom of 96-well micro-E-plate every 5 min for up to 23–32 h, 10^4^ tumor cells/well in complete medium were added to the plates. After 23–32 h, medium with or without previously titrated optimal concentrations of construct or substances were added. One μg/mL (final concentration) bsAb [HER2xCD3] or corresponding control constructs as well as 50 μM COX-1/2 inhibitor Indomethacin, 30 μM selective COX-2 inhibitor DuP697 (both from Tocris Bioscience, Bristol, UK), 2.5 mM selective COX-1 inhibitor valeryl salicylate (Cayman Chemical Company, MI, USA), 20 μM pan-caspase inhibitor zVAD-fmk (Bachem, Bubendorf, Switzerland) or 1 μg/mL PGE2 (Tocris Biosciences, Bristol, UK) were added in additional experiments 1 h before addition of γδ T cells. The bsAb [HER2xCD3] targets CD3-expressing T cells to HER2^+^ tumor cells (34). Impedance of the cells reflects changes in cellular parameters such as morphological changes (e.g., adherence, spreading), cell proliferation and cell death and is expressed as an arbitrary unit called cell index (CI). Since the initial adherence in different wells can differ slightly, the CI was normalized to 1 after the cells have reached the linear growth phase. After 23–32 h, Vδ1 and Vδ2 T cell lines with a previously titrated effector/target (E/T) ratio of 25:1 together with 12.5 U/mL IL-2 were added to the tumor cells. Loss of impedance of tumor cells is shown as decrease of the normalized CI due to Vδ1 T cell lines-induced lysis of the malignant cells. Different cancer cells were treated with 1% Triton X-100 (final concentration) as a positive control for killing. For the precise analysis of cytotoxicity, the cells were monitored every minute for the indicated time points. By using the RTCA software (version 2.0.0.1301 Copyright © 2004–2012 ACEA Biosciences Inc.) the raw data files were exported to Microsoft Excel [version 14.0.7128.5000 (32-bit)] for further calculation and described as follows. The mean of Triton-X-100 samples was calculated and defined as 100% lysis over 10 h after addition of T cells. The ratio of each sample (control- or TRAIL-R4-KD cells plus Vδ1 or Vδ2 T cells) to its appropriate control (identical control- or TRAIL-R4-KD without Vδ1 or Vδ2 T cells) was calculated and the ratio was normalized to maximal inducible lysis by Triton-X-100. In some experiments, percentage of lysis was calculated at 4 h after addition of T cells (Figures 2B, 7C). In other experiments, where an increase of impedance of tumor cells instead of a decrease in the presence of effector cells is shown (due to resistance against lysis), fold change in CI was calculated at 4 h after addition of T cells (Figures 3B, 8C). Time point zero was defined as first measurement after addition of T cells.

### Viability Assay

Fifteen thousand cells were seeded/96-well and incubated for 24 h. The cells were treated with 50 ng/mL recombinant human sTRAIL/Apo2L (#310-04, PeproTech, Germany) for 24 h. Cellular viability was detected using EZ4U (#BI-5000, Biomedica Medizinprodukte GmbH, Vienna) according to manufacturer's protocol.

### Enzyme-Linked Immunosorbent Assay

Ten thousand PDAC cells transfected with TRAIL-R4-specific shRNA (clone a and b) or scrambled shRNA (control) were seeded in 96-well flat bottom microtiter plates (Nunc, Wiesbaden, Germany). After 23 h, 1 μg/mL (final concentration) bsAb [HER2xCD3] or 50 μM Indomethacin or the combination of both were added 1 h before addition of Vδ1 T cell lines (E/T ratio: 25:1) supplemented with 12.5 U/mL rIL-2 for further 24 h. To quantify PGE2 released by PDAC cells alone or after coculture with Vδ1 T cell lines as well as granzyme B released by Vδ1 T cell lines cocultured with PDAC cells in the absence or presence of bsAb or COX1/2 inhibitor, supernatants were collected after incubation time and stored at −20°C until use. PGE2 was measured by Prostaglandin E2 Parameter Assay Kit (#SKGE004B) and human granzyme B by a sensitive sandwich ELISA (both from R&D System) in duplicates following the procedures outlined by the manufacturer.

### Statistics

Statistical analysis was assessed by One-way-Anova or Wilcoxon rank sum test using Graph Pad Prism (Graph Pad Software, Inc., La Jolla, CA, USA) or by a para-metrically *t-*test using (Microsoft Excel). The level of significance of all statistical tests was set at 5%. The Shapiro–Wilk test (Graph pad Prism) was used to determine the normal distribution of the various data from at least three independent biological replicates.

## Results

### Knockdown of TRAIL-R4 Reduces Sensitivity of Cancer Cells to γδ T Cell-Induced Cytotoxicity

Although γδ T cells lyse a broad range of different tumor cells, they are largely ineffective against several other tumor cells. Yet, the mechanisms behind this deficit are not completely understood. Beside granzyme B and perforin, γδ T cells can also utilize death receptor ligands, among them TRAIL, to mediate cytotoxicity against tumor cells (54, 55). TRAIL-R4 has been shown to be significantly up-regulated in PDAC tissue compared to non-malignant ducts from PDAC patients or non-cancer patients (27). Importantly, this receptor may act as a negative regulator of TRAIL receptor-mediated apoptosis in different tumor cells. Since our unpublished data showed the expression of TRAIL-R4 in different tumor cells, we wondered whether this receptor could at least be partially responsible for the establishment of a resistant phenotype of these cells to γδ T cell-induced cytotoxicity as we recently reported (48, 56).

**Figure 1:**
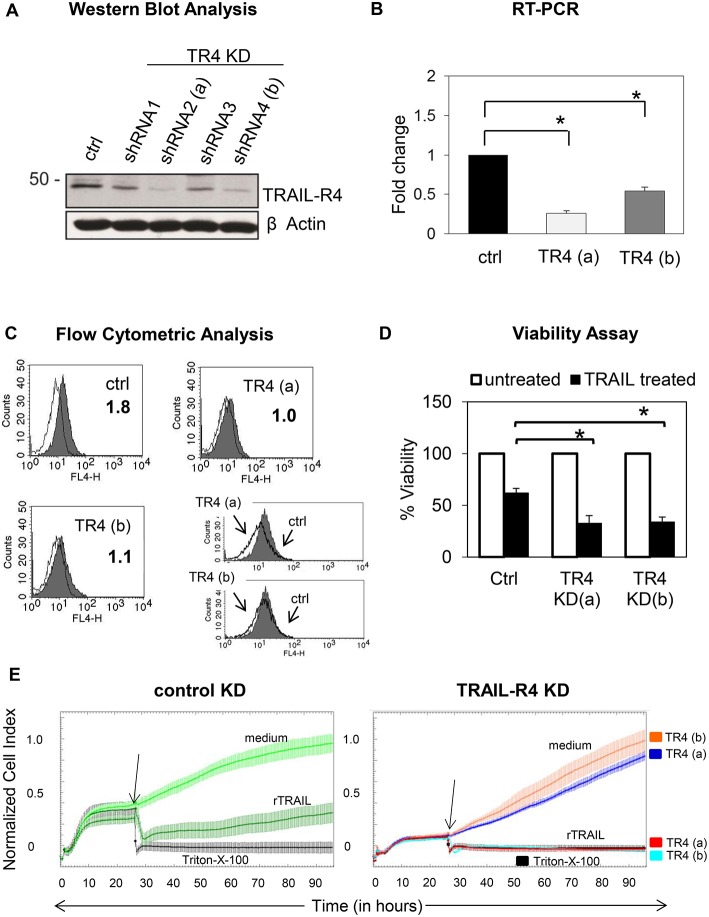
Characterization of control- and TRAIL-R4 knockdown Colo357 cells. **(A)** Colo357 cells were transduced with either control lentiviral shRNA or TRAIL-R4 specific shRNA to stably knockdown TRAIL-R4. The protein level of TRAIL-R4 was detected in the whole cell lysate of the transduced cells via western blot. Additionally the mRNA level of TRAIL-R4 in TRAIL-R4 KD clones [TR4 KD (a) and (b)] was measured via qPCR **(B)**, shown is the mean of three biological replicates. **(C)** Furthermore, cell surface expression of TRAIL-R4 in the TRAIL-R4-KD clones (a) and (b) (gray filled histograms; thin lines, isotype control) was detected by using flow cytometry. Shown is a representative histogram out of six biological replicates and an overlay of control- (gray filled histograms) vs. TR4-KD Colo357 cells (bold lines). **(D)** The viability of cells under TRAIL treatment (50 ng/mL) was determined by EZ4U assay. Shown is the mean of three biological replicates. Student *t-*test was done to determine significance (* < 0.05 *p-*value). **(E)** Ten thousand control- (light green line) or TRAIL-R4-knockdown (TR4-KD) Colo357 cells [TR4-KD (a), dark blue line; TR4-KD (b), orange line] were cultured in complete medium for 95 h on E-plates. The bottom of the E-plates were covered with electronic sensors that measured the impedance of these adherent tumor cells expressed as an arbitrary unit called cell index (CI) every 5 min. The arrows mark the addition of substances after 26 h as follows: medium [light green line for control KD Colo357 cells, dark blue line for TR4-KD- (a), orange line for TR4-KD (b) Colo357 cells], 100 ng/mL recombinant TRAIL [dark green line for control KD cells, red line for TR4-KD (a), and light blue line for TR 4 KD (b) cells] or Triton-X-100 to induce maximal lysis (black line). CI was then measured for additional 69 h. The loss of tumor cell impedance and thus a decrease of CI correlated with lysis of tumor cells. The average of triplicates and standard deviation were calculated; one representative experiment out of three is shown.

To address this issue, we first generated Colo357 and MDA-MB-231 cells with suppressed expression of TRAIL-R4 by stable transfection with TRAIL-R4-specific shRNA. All resulting TRAIL-R4-knockdown (KD) cell lines expressed lower levels of TRAIL-R4 than the corresponding control cells transfected with non-silencing shRNA (Figure 1A, Supplementary Figures 1A,B). Two of these obtained Colo357 KD cells and one of the MDA-MB-231 KD cells, which showed the most efficient knockdown of TRAIL-R4 [Figure 1A; TRAIL-R4-KD lines (a) and (b), Supplementary Figures 1A,B], were selected for further investigations. In all TRAIL-R4-KD cells, the expression of the other TRAIL-receptors was comparable (Figure 4B, Supplementary Figures 1A,B). Beside the clear decrease of the total cellular content of TRAIL-R4 protein (Figure 1A, Supplementary Figures 1A,B) and TRAIL-R4-mRNA (Figure 1B), these clones also showed, albeit less pronounced, diminished levels of this receptor at the cell surface (Figure 1C, data not shown).

For functional validation of the TRAIL-R4-KD cells, their sensitivity to TRAIL-mediated cell death was determined by two different methods, an EZ4U-Assay, which measures the mitochondrial metabolic activity (Figure 1D) and the Real Time Cell Analyzer (RTCA) system, which measures changes in cell adherence using the impedance technology (Figure 1E). Consistent with its known inhibitory function, knockdown of TRAIL-R4 clearly increased the sensitivity of cells to TRAIL-mediated apoptosis (Figures 1D,E).

**Figure 2:**
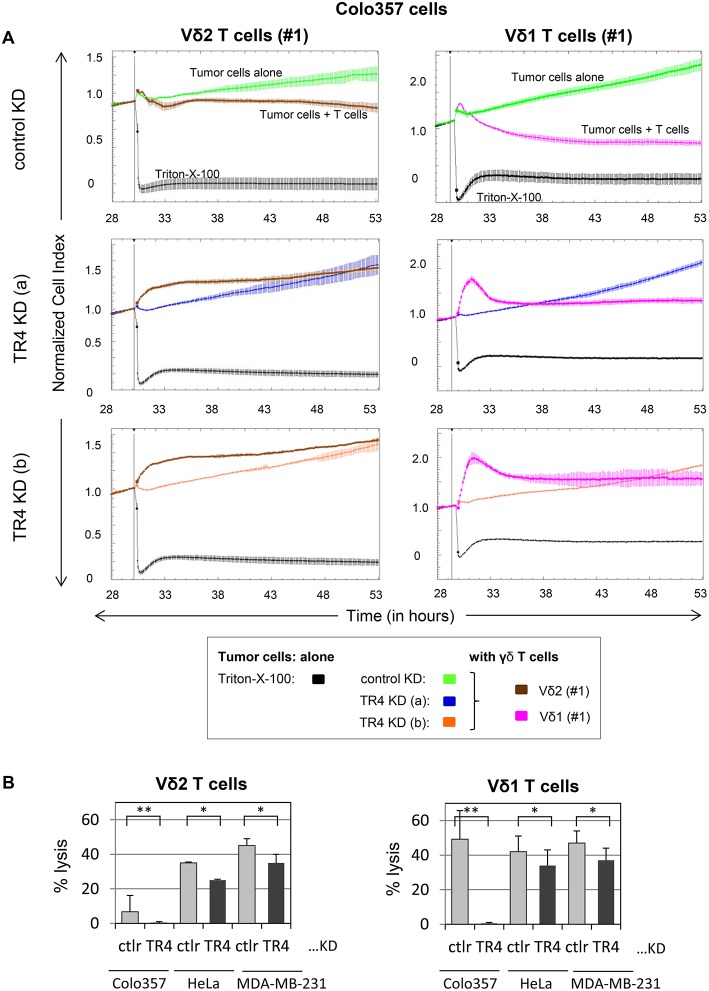
Enhanced Vδ1 T cell-mediated lysis of tumor cells compared to Vδ2 T cells is diminished by TRAIL-R4 knockdown. **(A)** After culturing 10^4^ control KD- (green lines) or two TRAIL-R4 KD Colo357 cells [TR4 KD (a), dark blue lines and TR4 KD (b), orange lines] in complete medium for 30 h, impedance of these adherent tumor cells expressed as cell index (CI) was measured every 5 min. The CI was normalized to 1 shortly before the addition of substances as follows: Triton-X-100 to induce maximal lysis (black line), Vδ2 γδ T cell line (brown lines) or Vδ1 γδ T cell line (pink lines) of healthy donor #1 (E/T ratio 25:1) with 50 IU/mL rIL-2. CI was then measured every minute for additional 23 h. The loss of tumor cell impedance and thus a decrease of CI correlated with lysis of tumor cells. The average of triplicates and standard deviation were calculated; one representative experiment out of three for Vδ2 γδ T cell lines and eight for Vδ1 γδ T cell lines is shown. **(B)** Ten thousand control KD (ctrl) or the indicated TRAIL-R4 (TR4) KD tumor cells were cultured under the same conditions as described under **(A)**, and also cocultured with Vδ2- or Vδ1 γδ T cell lines (E/T ratio 25:1) in the presence of 12.5 IU/mL rIL-2 in the RTCA. Percentage lysis was analyzed from RTCA data by calculating the normalized impedance of spontaneous lysis (cell growth of tumor cells in medium alone) in relation to the maximal lysis induced by 1% Triton-X-100. The mean of six (for Colo357 cells) to three (for the other tumor cells) individual samples cultured as triplicates plus standard deviation are shown 4 h after the addition of γδ T cells. Significances are shown as *P-*value; *P < 0.05 and **P < 0.01.

Next, we examined whether these cells are also more sensitive to γδ T cell-mediated cytotoxicity. For this purpose, we incubated the TRAIL-R4-KD cells or the control cells with different γδ T cell lines of different healthy donors and cancer patients and analyzed the lysis of tumor cells using RTCA system (Figure 2, Supplementary Figure 2). Here, in addition to Vδ2 γδ T cells, we also tested the killing capacity of Vδ1 γδ T cells, the cell population which we found to be mainly present in the tumor tissue (unpublished observation). After Colo357 cells were allowed to adhere for 24 h, the effector cells (>95% purity) were added and changes in Cell Index (CI) were monitored over 20 h.

**Figure 3:**
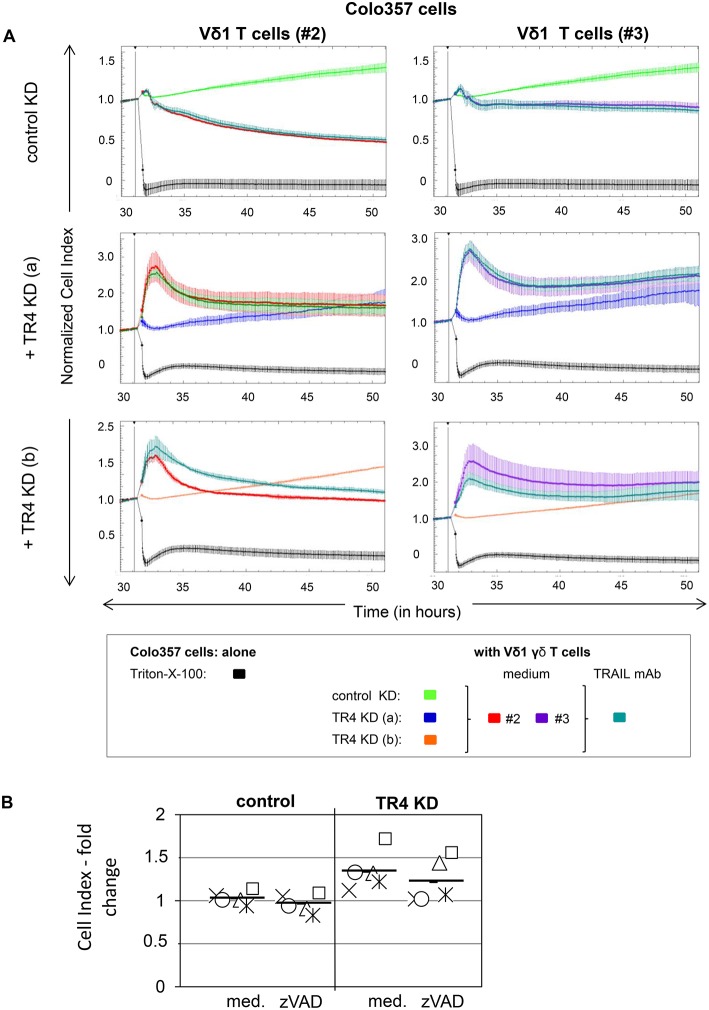
Neutralization of TRAIL does not reverse the inhibitory effects of the TRAIL-R4 KD in Colo357 cells on Vδ1 T cell-mediated cytotoxicity. **(A)** Ten thousand control KD—and TRAIL-R4 KD [TR4 KD (a) or TR4 KD (b)] Colo357 cells per well were cultured in complete medium overnight. Cell Index (CI) was analyzed in 5 min steps over ~ 32 h. After overnight adherence, Colo357 cells were cultured with additional complete medium [control: green, TR4 KD (a): blue dark or TR4 KD (b): orange line] or positive control Triton-X-100 (black line). After 32 h, Colo357 cells were cocultured with two Vδ1 γδ T cell lines of different donors (#2, red lines and #3, purple lines) with an E/T ratio of 25:1 and 12.5 IU/mL rIL-2 in the presence of medium (red or purple lines) or 1 μg/mL TRAIL mAb (dark green lines). Lysis of tumor cells was measured after normalization to 1 in one min steps for >18 h as indicated. The average of three replicates with SD is represented for each tumor cell line with effector cells of one representative healthy donor (#2) and one pancreatic cancer patient (#3) in independent experiments. **(B)** The culture conditions were similar to the ones described in **(A)** with the difference that only control KD—and TRAIL-R4 KD [TR4 KD (a)] Colo357 cells were applied as target cells. After 32 h, Colo357 cells were cocultured with five different Vδ1 γδ T cell lines of different donors with an E/T ratio of 25:1 and 12.5 IU/mL rIL-2 in the presence of medium or 20 μM zVAD-fmk. Each symbol represents a different donor. Black bars represent mean of the five independent experiments. Cytotoxicity was analyzed by Real-Time Cell Analyzer and fold change in Cell Index (CI) was calculated using formula as follows: $CI-Fold\;change=1-(\left(1-\left(\frac{S}{C}\right)*\left(\frac{1}{1-\frac{M}{C}}\right)\right)$; S, CI value of the sample; C, value of the medium control; M, Mean of CI value of Triton-X-100 sample.

RTCA analysis revealed that Colo357 cells were highly resistant to Vδ2 γδ T cell-induced cytotoxicity and less resistant but still not completely lysed by Vδ1 γδ T cells (Figure 2A). Surprisingly, knockdown of TRAIL-R4, instead of sensitizing the cells to γδ T cell-induced lysis, rendered these cells even more resistant. The acquired resistance of TRAIL-R4-KD cells toward γδ T cell-mediated cytotoxicity was confirmed by independent experiments performed with additional Vδ2- and Vδ1 γδ T cell lines (Figures 3, 7, 8, Supplementary Figure 2; for optimal quantification and simplified presentation in Figures 3B, 7, 8, cytotoxic capacity was calculated 4 hrs after addition of γδ T cell lines as a decrease in fold change of CI compared to the control sample without effector cells and to maximal lysis with Triton-X-100). Beside Colo357 and MDA-MB-231 cells, TRAIL-R4 was also transiently knocked-down via siRNA in HeLa cells (Supplementary Figure 1C). Importantly, all stable and transient TRAIL-R4 knockdown cells presented a significantly reduced sensitivity against γδ T cell-mediated cytotoxicity (Figure 2B). As shown in Supplementary Figure 1C, TRAIL-R1 was slightly regulated, whereas TRAIL-R2/-R3 were not affected by knockdown of TRAIL-R4 in HeLa cells. Of note, the results showing the potential regulatory role of tumor cell-expressed TRAIL-R4 on γδ T cell-induced cytotoxicity were similar for both γδ T cell-populations-derived cell lines. The importance of TRAIL-R4 as a regulatory protein was underlined by results demonstrating an enhanced γδ T cell-mediated cytotoxicity toward TRAIL-R4 knockin (KI) HeLa cells (Supplementary Figure 3). Since Vδ1 γδ T cells comprise the main population in PDAC patients (34), we focused our study on these γδ T cell-subset. As TRAIL-R4-KD cells were very sensitive to TRAIL-treatment (Figures 1D,E), the diminished sensitivity of these cells to γδ T cells-mediated cytotoxicity suggested that TRAIL did not play a pivotal role in killing of tumor cells by γδ T cells. In accordance, neutralization of TRAIL via anti-TRAIL antibody or TRAIL-R2-Fc showed no effect on lysis of either control or of TRAIL-R4-KD cells (Figure 3, and data not shown). A negligible role of the TRAIL-mediated lysis of control- or TRAIL-R4-KD Colo357 cells by γδ T cells was confirmed by additional independent experiments. These results showed an insignificant effect on the Colo357 killing by γδ T cells in the presence of pan-caspase inhibitor zVAD-fmk (Figure 3B). In addition, knockdown of death-inducing TRAIL receptors, TRAIL-R1 or TRAIL-R2 in Colo357 cells, had minimal (TRAIL-R1) or no (TRAIL-R2) influence on the sensitivity of these cells to Vδ1 T cell-mediated cytotoxicity (Supplementary Figure 4).

Unlike Colo357 and MDA-MB-231 cells, PancTuI cells showed high sensitivity to the lysis by Vδ1 T cells in both control and TRAIL-R4-KD shRNA cells (Supplementary Figure 5). In contrast to the cytotoxicity of Vδ1 T cells, the Vδ2 T cell-mediated cytotoxicity against PancTuI cells was very weak in the absence of enhancers of γδ T cell-cytotoxicity such as bsAb (48). Coculture of PancTuI cells with Vδ1 γδ T cells resulted in their lysis which was not affected by either neutralization of TRAIL or knockdown of TRAIL-R1 or TRAIL-R2 (Supplementary Figures 5, 6).

### Knockdown of TRAIL-R4 Enhances Expression of COX-1 and COX-2 and the Secretion of PGE2

**Figure 4:**
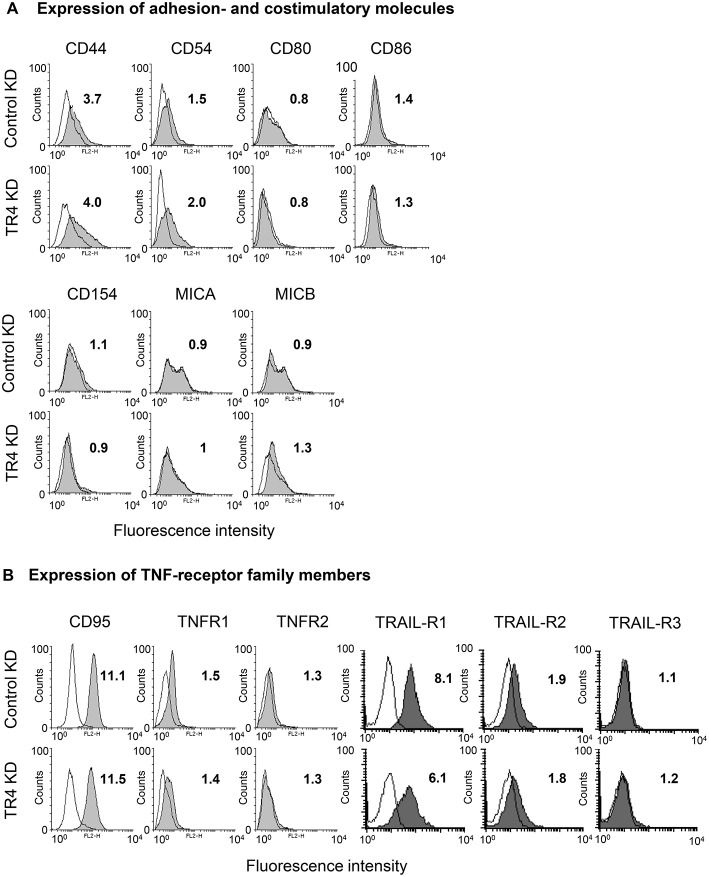
Impact of TRAIL-R4 KD on the expression of adhesion- and costimulatory molecules as well as TNF-receptor family members on Colo357 cells. Flow cytometric analysis of the indicated **(A)** adhesion- and costimulatory surface receptors and **(B)** CD95, TNF-R1, TNF-R2, TRAIL-R1, -R2, and -R3 expressed on control KD - and TRAIL-R4 KD (TR4 KD) Colo357 cells determined by staining the cells with the appropriate monoclonal antibodies (gray histograms) and appropriate isotype controls (open black lines). The expression was calculated as the fold change of the median of specific antibody signal relative to the isotype control. Median of fold changes of one representative histogram out of four independent experiments is shown.

As cytotoxic activity of Vδ1 T cells against TRAILR4-KD tumor cells—except for PancTuI cells—was significantly impaired, we asked whether the down-regulation of TRAIL-R4 in these cells led to an altered expression of molecules involved in T cell-mediated cytotoxicity. The adhesion molecule CD44 was reported to be highly expressed on different tumor cells and to suppress T cell-mediated immune responses (57, 58). Our data revealed that CD44 was weakly expressed on Colo357 cells and only slightly up-regulated in TRAIL-R4-KD cells (Figure 4A). The interaction of CD54 and its corresponding ligand CD11a/CD18 expressed on γδ T cells is responsible and therefore essential for triggering cytotoxic function in γδ T cells (40, 59). However, CD54 was rather up-regulated than down-regulated in TRAIL-R4-KD cells (Figure 4A). In addition, the costimulatory receptors CD80/CD86, CD154, or MICA/MICB were very weakly expressed on Colo357 cells and did not significantly differ between control- and TRAIL-R4-KD Colo357 cells (Figure 4A). Furthermore, the expression of death receptors CD95, TNFR1, TRAIL-R1, and TRAIL-R2 as well as of TNFR2 and TRAIL-R3 did not significantly differ between control- and TRAIL-R4-KD cells (Figure 4B, Supplementary Figure 1C). In conclusion, these data suggest that mechanisms other than changes in expression of adhesion- or co-stimulatory molecules and cell death receptors account for the enhanced resistance of TRAIL-R4-KD cells to γδ T cell-induced cytotoxicity.

**Figure 5:**
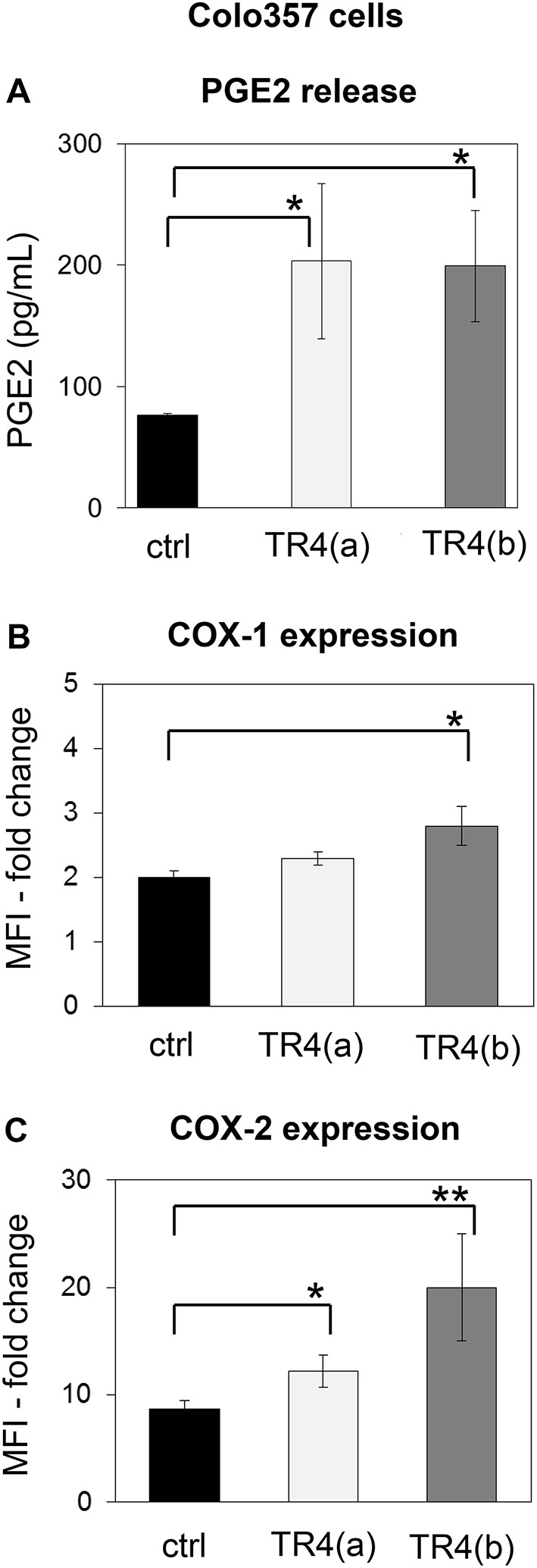
Enhanced PGE2 release and COX-expression of TRAIL-R4 knockdown Colo357 cells in comparison to control cells. **(A)** PGE2 release was determined in the supernatant of control KD–and two different TRAIL-R (TR) 4 KD (a) or (b)—Colo357 cells after overnight culture. **(B,C)** In parallel, cells were intracellularly stained with anti-COX-1 and anti-COX-2 mAb and analyzed by flow cytometry. The expression is shown as fold change in median fluorescence intensity (MFI) relative to isotype control. Bars represent mean ± SD of three independent experiments. Significances are shown as *P*-value; * = *P* < 0.05 and ** = *P* < 0.01.

One prominent defense mechanism that tumor cells developed to counteract the cytotoxic activity of T cells is the secretion of PGE2, which can diminish both the cytotoxicity and the proliferation of T cells (48, 60). Interestingly, we found that knockdown of TRAIL-R4 in Colo357 cells resulted in a significant enhancement of PGE2 secretion (Figure 5A). The PGE2 synthesis is mainly regulated by two enzymes, the constitutively active COX-1 and an inducible enzyme COX-2 (61, 62). To study whether the knockdown of TRAIL-R4 impacts on COX-1- and COX-2-expression, we compared the intracellular expression of both enzymes in control- and TRAIL-R4-KD cells by flow cytometry. We found that both, control and TRAIL-R4-KD cells expressed COX-1 as well as COX-2 (Figures 5B,C). However, TRAIL-R4-KD cells showed enhanced levels of both enzymes. Particularly COX-2 expression was strongly and significantly up-regulated in TRAIL-R4-KD cells compared to control cells (Figure 5C, Supplementary Figures 7A,B). In agreement with our previously published data, PancTuI cells do not express COX-2 [(48) and Supplementary Figure 7A], whereas MDA-MB-231 cells express low levels of COX-2 (Supplementary Figure 7B). Of note, knockdown of TRAIL-R4 in PancTuI cells did not change either their sensitivity to Vδ1 T cell-mediated cytotoxicity or the COX-2 expression (Supplementary Figure 5), consistent with the postulated role of COX-2 in the inhibition of Vδ1 T cells-induced cytotoxicity toward Colo357 TRAIL-R4-KD cells. Knockdown of TRAIL-R4 in MDA-MB-231 cells resulted in a slight increase of COX-2 expression which explains the moderate resistance of TRAIL-R4-KD cells toward γδ T cell-mediated lysis compared to TRAIL-R4-KD Colo357 cells (Figure 2B, Supplementary Figure 7B).

**Figure 6:**
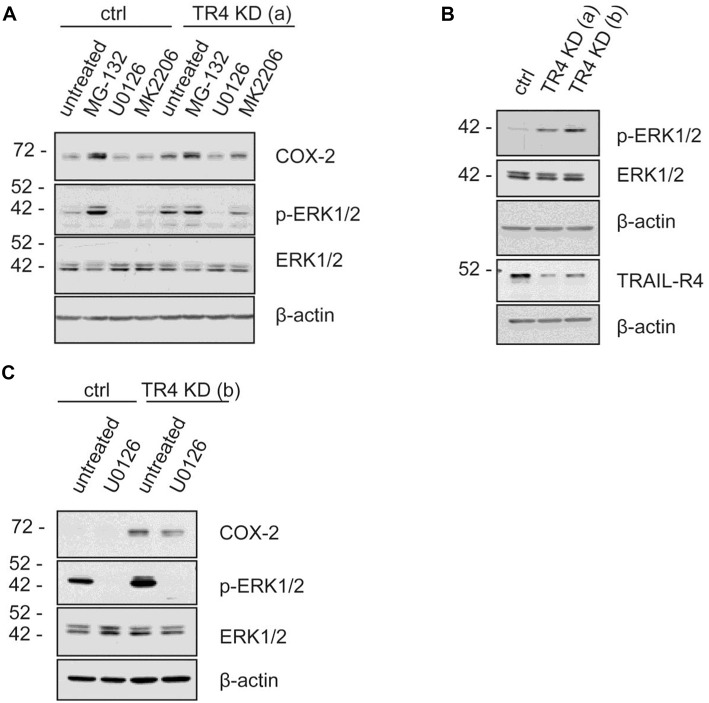
MAP Kinases ERK1/ERK2 regulate COX-2 level in Colo357 cells. Control KD- as well as TRAIL-R4 KD [TR4 KD (a)] Colo357 cells were treated with four different inhibitors [MG-132 (proteasome inhibitor), U0126 (MEK inhibitor), MK2206 (AKT1,2,3 inhibitor)] for 8 h. **(A)** The protein levels of COX-2, phospho-ERK1/2 and ERK were detected in whole cell lysates via western blot. **(B)** The basal level of the different proteins in both TR4 KD- (a, b) and control Colo357 cells is shown. **(C)** The correlation between COX-2, phospho-ERK1/2 and ERK1/2 in control KD and TR4 KD (b) cells is presented.

To study the putative mechanisms responsible for the observed increased expression of COX-2 in TRAIL-R4-KD Colo357 cells, we treated these cells as well as control cells with inhibitors of various signal transduction pathways known to play a role in TRAIL-R-induced signaling and determined their impact on COX-2 expression. Consistent with the flow cytometry data (Figure 5C), Western blot analyses of whole cell lysates showed marked upregulation of the cellular levels of COX-2 in TRAIL-R4-KD cells compared to the control cells (Figures 6A,B). The inhibition of AKT by MK2206 did not change the COX-2 level, whereas the inhibition of proteasome by MG-132 strongly upregulated the expression of COX-2 in both control- and TRAIL-R4-KD cells. Importantly, inhibition of MAP-kinases ERK1/ERK2 by U0126 severely reduced the amounts of COX-2 in TRAIL-R4-KD cells (Figures 6A,C). In agreement, Western blot analyses performed using phosphorylation/activity status-detecting antibodies revealed that TRAIL-R4-KD cells are characterized by strongly up-regulated activity of ERK1/ERK2 with no observed changes in the overall cellular levels of these kinases (Figures 6A–C). In contrast, HeLa TRAIL-R4-KI cell line showed a lower activity of ERK1/ERK2 (Supplementary Figure 7D).

**Figure 7:**
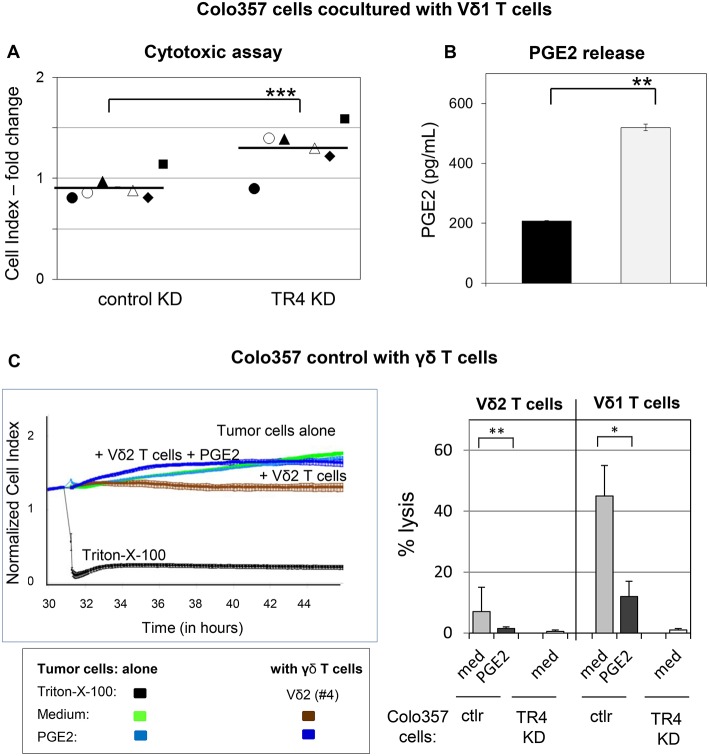
Decreased Vδ1 T cell-cytotoxicity against TRAIL-R4 knockdown Colo357 cells. Ten thousand control- and TRAIL-R (TR) 4 KD Colo357 cells were cocultured with Vδ1 T cell lines coexpressing a Vγ2, 3, or 4 chain (closed symbol in **A**) or a Vγ8 chain (open symbol in **A**) with an E/T ratio of 25:1 and a final concentration of 12.5 U/mL IL-2 for 4 h. Thereafter, **(A)** cytotoxicity was analyzed by Real-Time Cell Analyzer and **(B)** PGE2 release out of the supernatant by ELISA. Fold change in Cell Index (CI) was calculated using formula as follows: $CI-Fold\;change=1-(\left(1-\left(\frac{S}{C}\right)*\left(\frac{1}{1-\frac{M}{C}}\right)\right)$; S, CI value of the sample; C, vale of the medium control; M, Mean of CI value of Triton-X-100 sample. Black lines indicate mean of six independent experiments. Significances are shown as *P*-value; ** = *P* < 0.01 and *** = *P* < 0.001. **(C)** After culturing 10^4^ Colo357 cells (green line) in complete medium for 30 h, impedance of these adherent tumor cells expressed as CI was measured in 5 min steps. The CI was normalized to 1 shortly before the addition of substances as follows: Triton-X-100 to induce maximal lysis (black line), medium (green line), 1 μg/mL PGE2 (light blue line), Vδ2 γδ T cell line (brown line) or Vδ2 γδ T cell line plus PGE2 (dark blue lines) with an E/T ratio of 25:1 in the presence of 12.5 IU/mL rIL-2. CI was then measured in 1 min steps over additional 26 h. The loss of tumor cell impedance and thus a decrease of CI correlated with lysis of tumor cells. The average of triplicates and standard deviation were calculated; one representative experiment. Several replications of the experiments using four different Vδ2 T cell lines and five different Vδ1 T cell lines of different donors in independent experiments were performed (right panel). The cytotoxicity of γδ T cell lines against the indicated tumor cells in the presence of medium or PGE2 was calculated 4 h after addition of γδ T cell lines. The percentage of specific lysis was calculated by comparing measured samples to control sample without effector cells and maximal lysis. Statistical analysis was performed by *t-*test. Significances are shown as *P-*value; *P < 0.05 and **P < 0.01.

A possible explanation for the weak lysis of TRAIL-R4-KD cells in comparison to control Colo357 cells by γδ T cell lines could be due to an enhanced PGE2 release by TRAIL-R4-KD cells as already indicated in Figure 4A. To proof this hypothesis, we cocultured Vδ1 T cell lines from different healthy donors (*n* = 4) and PDAC patients (*n* = 2) with either control- or TRAIL-R4-KD cells and compared the cytotoxic potential of Vδ1 T cell lines toward both cell lines concomitantly measuring the PGE2 release (Figures 7A,B). All applied Vδ1 T cell lines induced a partial lysis of control Colo357 cells, while TRAIL-R4-KD Colo357 cells were not lysed, as demonstrated by significantly higher CI values compared to control cells (Figure 7A). These results were independent of the Vδ1 T cell line used and the co-expression of specific Vγ-chains (Figure 7A, closed symbols: Vδ1 T cells co-expressing Vγ2, 3 or 4 chain, open symbols: Vδ1 Vγ8 T cells), indicating a lower capacity of different Vδ1 T cell-subsets to lyse TRAIL-R4-KD cells compared to control cells. Interestingly, this lower capacity was accompanied by a significantly enhanced PGE2 release by TRAIL-R4-KD cells cocultured with Vδ1 T cell lines in comparison to control Colo357 cells (Figure 7B). Our results were confirmed by additional independent experiments demonstrating that the addition of exogenous PGE2 decreased the Vδ2- as well as Vδ1 γδ T cell-mediated lysis of control Colo357 cells to a similar level as observed with TRAIL-R4-KD (Figure 7C).

**Figure 8:**
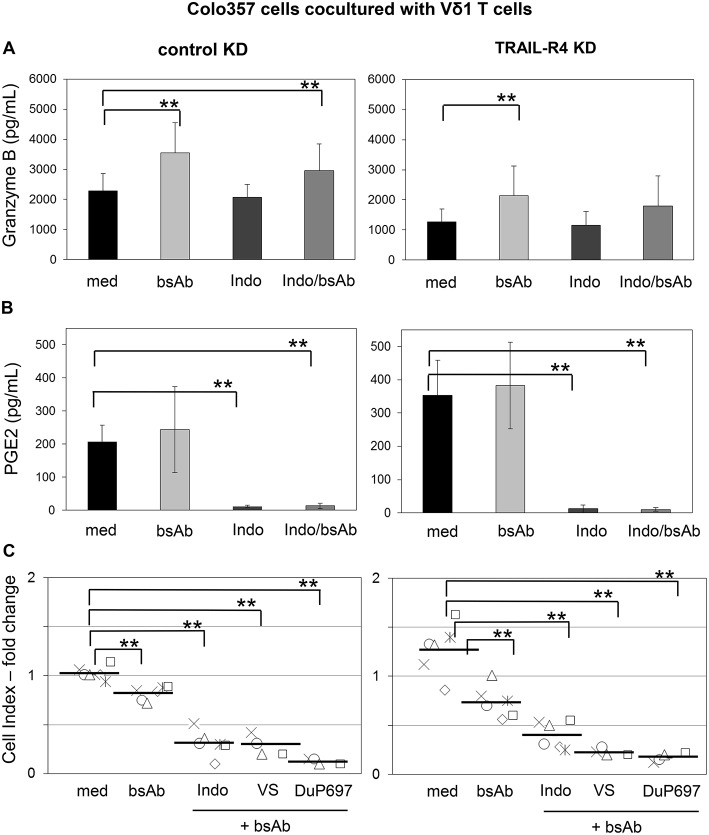
Increased Vδ1 T cell-cytotoxicity against Colo357 cells by bsAb [HER2xCD3] is further enhanced by COX inhibitors. Ten thousand control- or TRAIL-R (TR) 4 KD Colo357 cells were incubated for 24 h in 96-well E-plates in medium. After 23 h, a final concentration of 1 μg/mL bsAb [HER2xCD3] or 50 μM Indomethacin alone or in combination or alternatively in combination with 30 μM DuP697 or 2.5 mM valeryl salicylate as indicated were added. After 24 h, Vδ1 T cell lines of six different donors with an E/T ratio of 25:1 and a final concentration of 12.5 U/mL IL-2 were cocultured. **(A)** The release of granzyme B or **(B)** PGE2 were measured after 24 h by ELISA and **(C)** Vδ1 T cell-mediated cytotoxicity against Colo357 cells by RTCA shown as decrease in Cell Index (CI) fold change calculated with the formula mentioned in Figure 7. Black bars represent mean of experiments. Significances are shown as *P*-value; ** = *P* < 0.01.

Recently, we demonstrated that the Vδ2-expressing γδ T cells exert their cytotoxic activity against PDAC cells mainly via granzyme B released from cytolytic granules (34). The granzyme B release can be drastically enhanced by an addition of bsAb leading to a strong enhancement of the Vδ2 T cell-cytotoxicity (34, 53). Since cells with TRAIL-R4-KD were almost completely refractory to cytotoxic activity of Vδ1 T cells, we next investigated whether TRAIL-R4-KD cells could negatively influence the secretion of granzyme B by Vδ1 T cells. Indeed, we found that Vδ1 T cells released lower amounts of granzyme B when cocultured with TRAIL-R4-KD than with control cells (Figure 8A, med, left, and right panel). The addition of bsAb [HER2xCD3] significantly enhanced the granzyme B release and as a consequence the Vδ1 T cell-mediated lysis of control cells and TRAIL-R4-KD cells (Figures 8A,C). Since the inhibition of granzyme B release by Vδ1 T cells cocultured with TRAIL-R4-KD cells was accompanied by an enhanced PGE2 release of the latter, we additionally analyzed the effects of COX-1/2 inhibitor Indomethacin on granzyme B and PGE2 release. While Indomethacin significantly reduced the PGE2 release by control- as well as by TRAIL-R4-KD Colo357 cells, it had no impact on the granzyme B release by any of the cells. Conversely, while the addition of bsAb clearly enhanced the granzyme B release by Vδ1 T cells, it did not significantly alter PGE2 release by the tumor cells (Figure 8B). The latter could be explained by the observation that the treatment of Colo357 cells with bsAb did not alter the expression of neither COX-1 nor COX-2 in tumor cells (Supplementary Figure 7C). Importantly, concomitant administration of bsAb and Indomethacin enhanced the Vδ1 T cell-cytotoxicity toward control- and TRAIL-R4-KD cells more effectively than the separate administration of either substance alone (Figure 8C and data not shown). The application of bsAb together with the selective inhibitors of either COX-1 (valeryl salicylate -VS-) or COX-2 (DuP697) strongly increased the sensitivity of both control and TRAIL-R4-KD cells toward Vδ1 T cell-lysis. However, these effects were more pronounced in TRAIL-R4-KD cells than in control Colo357 cells suggesting an enhanced release of PGE2 by these cells constitute an important defense mechanism (Figure 8C). These data were confirmed using HeLa and MDA-MB-231 TRAIL-R4-KD cells which in combination with bsAb showed similar tendency (data not shown).

Taken together, we demonstrated that γδ T cell-mediated lysis of COX-2 expressing tumor cells is regulated by TRAIL-R4. Knockdown of this receptor enhanced the synthesis and release of PGE2 by the tumor cells and reduced the release of granzyme B by cocultured Vδ1 T cells thereby inhibiting Vδ1 T cell-cytotoxicity. The application of bsAb which enhanced γδ T cell-cytotoxicity and granzyme B release together with COX inhibitors restored the sensitivity of Colo357 cells against γδ T cell-mediated cytotoxicity.

## Discussion

In this study, we demonstrated that TRAIL-R4 expressed by tumor cells regulates the cytotoxic activity of γδ T cells.

γδ T cells are attractive effector cells for T cell-based immunotherapy. Although clearly both Vδ1 and Vδ2 T cells are important for tumor immune surveillance, so far most of the studies have been dedicated to Vδ2 T cells. The preferential interest in Vδ2 T cells can be explained by (i) well-characterized antigens, (ii) the ease to expand these T cells *in vitro* under good manufacturing practice conditions for adoptive transfer, (iii) the application of licensed drugs such as aminobisphosphonates and IL-2 to activate these cells *in vivo*, (iv) availability of bispecific antibodies as enhancer for Vγ9Vδ2 γδ T cell-mediated lysis of tumor cells and (v) high plasticity of Vδ2 T cells by combining the features of both innate and adaptive immunity [(30, 63–68) for review].

In contrast, the functions of Vδ1 T cells are less well-understood. Importantly, however, these cells are enriched in the peripheral blood of PDAC patients (34) and infiltrate malignant pancreatic and ovarian tissues (unpublished observation). Our study revealed a superior Vδ1 T cell-cytotoxicity compared to Vδ2 T cell-cytotoxicity from the same donors against PGE2-secreting cancer cells. We also found that the cytotoxic activity of these cells is less prone to inhibition by PGE2, added exogenously to the coculture of Vδ1 T cells with tumor cells, than Vδ2 T cells. The difference of PGE2-effects on cytotoxicity of Vδ1- and Vδ2 T cells could be due to a weaker expression of prostaglandin E2 (PTGER2) and E4 (PTGER4) receptors on Vδ1 T cells than on Vδ2 T cells (data not shown). However, our study demonstrated that even though these receptors are weakly expressed, their levels are sufficient to negatively influence Vδ1 T cell-cytotoxicity against cancer cells which highly express the PG-synthetase COX. An autocrine PGE2 effect on PDAC cells (69) can be excluded due to the lack of prostaglandin receptor expression on these tumor cells (data not shown). Ligation of PGE2 with E2 and E4 receptors on γδ T cells initiates activation of the transcription factor cAMP responsive element binding (CREB) and nuclear factor κB (NF-κB) which results in the release of IFN-γ and TNF-α (48, 61, 70). Both cytokines are produced by the different γδ T cell subsets after activation and are able to enhance intracellular COX-2 expression in mesenchymal stem cells and in cancer cells as recently described by us and others for Vδ2 T cells (48, 71). Aside from IFN-γ and TNF-α, cytotoxic mediators such as granzymes and TRAIL are produced by γδ T cell-subsets and used for killing a broad range of tumor cells (34, 54, 55, 72). Particularly, the involvement of soluble TRAIL in the lysis of lung- and colon- and breast cancer cells by Vδ2 T cells was reported recently (54, 72–74).

Interestingly, in addition to the cell death induction, TRAIL has been shown to upregulate COX-1 activity and PGE2 secretion in cancer cells (75). Therefore, we were interested to know whether the TRAIL/TRAIL-R system might play a role in the sensitivity of tumor cells toward γδ T cell-cytotoxicity.

We found that neither the knockdown of TRAIL death receptors in PDAC cells (e.g., PancTuI- and Colo357 cells) nor the neutralization of TRAIL significantly impaired the killing potential of Vδ1 T cells. This suggests a minor role of TRAIL in the cytotoxic activity of these cells against the studied PDAC cells. In agreement, knockdown of anti-apoptotic TRAIL-R4 in some tumor cell lines did not enhance γδ T cell-mediated cytotoxicity, while strongly sensitized these cells to the treatment with recombinant TRAIL. Unexpectedly, however, it rendered these cells even more resistant to both Vδ2- and Vδ1 T cell-mediated cytotoxicity, independently of the co-expressed Vγ-chain, which underlines the observation that all γδ T cell-subsets are affected. These effects were associated with a reduced release of granzyme B by γδ T cells and an enhanced PGE2-production by TRAIL-R4-KD. The latter could be explained by the strong intracellular up-regulation of COX-2 expression in TRAIL-R4-KD cells and by an enhancement of expressed COX-1 in these cells; both cyclooxygenases are essential enzymes in the PGE2-synthesis. The expression of COX-2 is regulated by various signal transduction pathways including MAP Kinases ERK1/ERK2 (76). Importantly, we found that ERK-activity was strongly increased in TRAIL-R4-KD cells and its inhibition reduced the level of COX-2 in these cells to the level observed in the control. This suggests that increased ERK-activity is responsible for the upregulation of COX-2 expression in TRAIL-R4-KD cells. The reason why inhibition of TRAIL-R4 results in an increased ERK-activity is yet to be elucidated. Previously, we have shown that stimulation of Colo357 cells with TRAIL induces ERK-activity and this process requires the activity of apoptotic caspases (77, 78). Such caspase-dependent TRAIL-R-mediated induction of ERK-activity was reported also in other tumor cells (1). As many other tumor cells, Colo357 cells express small amounts of TRAIL (data not shown). It is therefore likely that the binding of tumor cell-derived TRAIL in TRAIL-R4-KD cells to TRAIL-R1/R2 results in a constitutively increased activity of caspases, which is too weak to induce cell death, but strong enough to activate ERK. In agreement, our unpublished data revealed that inhibition of caspases by zVAD-fmk in Colo357 TRAIL-R4-KD cells decreased COX-2 expression. However, the coculture of these cells with γδ T cells, in the presence of zVAD-fmk, did not significantly affect the tumor cell lysis. Interestingly, coculture of Colo357 cells with Vδ1 T cells enhanced PGE2 secretion, and this effect was more pronounced in cells with knockdown of TRAIL-R4 than in the control cells. In contrast to the obviously negligible role of TRAIL on Vδ1 T cell-cytotoxicity, arguably this might point to the role of TRAIL secreted by Vδ1 T cells on the enhancement of resistance of the studied tumor cells, especially those cells with TRAIL-R4-KD. Thus, small amounts of TRAIL secreted by Vδ1 T cells might slightly induce caspase-activity, again too weak to induce cell death yet strong enough to activate ERK leading to increased expression of COX-2 and consequently increased PGE2 production.

Consistent with the postulated role of COX-2 in the regulation of Vδ1 T cell-cytotoxicity toward control and TRAIL-R4-KD or TRAIL-R4-KI cells, we found that blocking of COX-2-activity by selective COX-2 inhibitor DuP697 rendered Colo357 cells sensitive to Vδ1 T cell-mediated cytotoxicity.

In addition to COX-2, COX-1 seems also to play a role in TRAIL-R4-KD-induced inhibition of Vδ1 T cell-cytotoxicity, as the selective COX-1 inhibitor valeryl salicylate also increased the lysis of Colo357 cells and reduced PGE2 secretion. COX-1 has been shown to be up-regulated by TRAIL and this effect was accompanied by activation of caspases and/or NFκB and led to a significant increase in PGE2 (69, 75, 79, 80). Inhibition of PGE2 synthesis represents a therapeutic aim in treatment of many disorders, among them cancer (81–83).

Since COX-1 is present in all tissues, COX-1 inhibition would affect the whole body. Therefore, the development of PGE2-inhibitors for clinical usage is focused on COX-2. DuP697 was the paradigm for the design of further COX-2 inhibitors such as Celecoxib which are included in clinical trials combined with other therapies [https://clinicaltrials.gov: COX-2 inhibitors (81–83)]. In addition to enhanced PGE2-secretion, TRAIL-R4-KD cells inhibited granzyme B release by γδ T cells. While COX-inhibitors could suppress PGE2 release by tumor cells, they did not increase the secretion of granzyme B by cytotoxic cells. Importantly, the concomitant usage of COX2-inhibitor with bsAb completely restored the killing capacity of γδ T cells toward TRAIL-R4-KD cells.

BsAb are a class of targeted biologics which are of great interest in cancer immunotherapy to target effector cells to the tumor site (84, 85). In this context, bsAb [HER2xCD3] which targets CD3-expressing Vδ1 T cells to HER2-expressing tumor cells seems to be very effective (34, 56). γδ T cells have raised substantial interest for immunotherapy based on their capacity to kill tumor cells, in a HLA-independent manner, which are often described to be resistant to radio- and chemotherapy (34, 53, 86, 87). A combined therapy of COX-2 inhibitors together with bsAb could be an attractive therapeutic option for COX-2 expressing tumors with downregulated expression of TRAIL-R4.

Our data suggests that TRAIL-R4 might play a decisive role in the immune surveillance. This could explain the observed downregulation of this receptor in different tumors which in some cases correlates with poor patients' prognosis (22, 28, 29, 88, 89). For PDAC, our own unpublished data revealed that TRAIL-R4 is almost exclusively localized to intracellular compartments instead of the plasma membrane. This could support the proposed scenario that TRAIL present in the tumor microenvironment, in the absence of plasma membrane expressed anti-apoptotic TRAIL-R4, would enhance TRAIL-R1/-R2-mediated COX2 expression thereby inhibiting γδ T cell activity.

Further studies are necessary to prove the generality of our findings. However, the previously unrecognized function of TRAIL-R4 in shaping the immune response strongly demands future research on the regulatory role of this receptor particularly in context of the other TRAIL receptors.

## Data Availability

The raw data supporting the conclusions of this manuscript will be made available by the authors, without undue reservation, to any qualified researcher.

## Ethics Statement

PBMC were isolated from leukocyte concentrates obtained from healthy blood donors and provided by the Department of Transfusion Medicine of the University Hospital Schleswig-Holstein (UKSH) in Kiel, Germany. In addition, heparinized blood from PDAC patients was obtained from the Department of General Surgery of the municipal hospital. In accordance with the Declaration of Helsinki, written informed consent was obtained from all donors, and the research was approved by the relevant institutional review boards (ethic committee of the Medical Faculty of the CAU Kiel, code number: D445/18).

## Author Contributions

DT, CG, H-HO, J-PG, and DW performed experiments. DT, CG, H-HO, and J-PG helped to design the study. MP designed and provided the bispecific antibodies. DT, CG, and H-HO wrote parts of the manuscript. TB provided blood from pancreatic cancer patients and contributed to the discussion. DK and MP contributed to the discussion and the composition of the manuscript. AT and DW designed the project and wrote and finalized the manuscript.

### Conflict of Interest Statement

The authors declare that the research was conducted in the absence of any commercial or financial relationships that could be construed as a potential conflict of interest.

## Abbreviations

bsAb: bispecific antibody
COX: cyclooxygenase
mAb: monoclonal antibody
MICA: MHC class-I related chain A
PDAC: pancreatic ductal adenocarcinoma
PG: prostaglandin
RTCA: Real Time Cell Analyzer
TCR, T cell receptor TRAIL: TNF-related apoptosis inducing ligand
PBMC: peripheral blood mononuclear cells.

## References

[1] Azijli K Weyhenmeyer B Peters GJ de JS Kruyt FA. Non-canonical kinase signaling by the death ligand TRAIL in cancer cells: discord in the death receptor family. Cell Death Differ. (2013) 20:858–68. 10.1038/cdd.2013.28 23579241 PMC3679459

[2] Degli-Esposti MA Dougall WC Smolak PJ Waugh JY Smith CA Goodwin RG. The novel receptor TRAIL-R4 induces NF-kappaB and protects against TRAIL-mediated apoptosis, yet retains an incomplete death domain. Immunity. (1997) 7:813–20. 10.1016/S1074-7613(00)80399-4 9430226

[3] Degli-Esposti MA Smolak PJ Walczak H Waugh J Huang CP DuBose RF. Cloning and characterization of TRAIL-R3, a novel member of the emerging TRAIL receptor family. J Exp Med. (1997) 186:1165–70. 10.1084/jem.186.7.1165 9314565 PMC2199077

[4] Pan G Ni J Wei YF Yu G Gentz R Dixit VM. An antagonist decoy receptor and a death domain-containing receptor for TRAIL. Science. (1997) 277:815–8. 10.1126/science.277.5327.815 9242610

[5] von KS Montinaro A Walczak H Exploring the TRAILs less travelled: TRAIL in cancer biology and therapy. Nat Rev Cancer. (2017) 17:352–66. 10.1038/nrc.2017.28 28536452

[6] Walczak H Degli-Esposti MA Johnson RS Smolak PJ Waugh JY Boiani N. TRAIL-R2: a novel apoptosis-mediating receptor for TRAIL. EMBO J. (1997) 16:5386–97. 10.1093/emboj/16.17.5386 9311998 PMC1170170

[7] Bertsch U Roder C Kalthoff H Trauzold A. Compartmentalization of TNF-related apoptosis-inducing ligand (TRAIL) death receptor functions: emerging role of nuclear TRAIL-R2. Cell Death Dis. (2014) 5:e1390. 10.1038/cddis.2014.351 25165876 PMC4454323

[8] Lemke J von KS Zinngrebe J Walczak H. Getting TRAIL back on track for cancer therapy. Cell Death Differ. (2014) 21:1350–64. 10.1038/cdd.2014.81 24948009 PMC4131183

[9] Hoogwater FJ Nijkamp MW Smakman N Steller EJ Emmink BL Westendorp BF. Oncogenic K-Ras turns death receptors into metastasis-promoting receptors in human and mouse colorectal cancer cells. Gastroenterology. (2010) 138:2357–67. 10.1053/j.gastro.2010.02.046 20188103

[10] Trauzold A Wermann H Arlt A Schutze S Schafer H Oestern S. CD95 and TRAIL receptor-mediated activation of protein kinase C and NF-kappaB contributes to apoptosis resistance in ductal pancreatic adenocarcinoma cells. Oncogene. (2001) 20:4258–69. 10.1038/sj.onc.1204559 11464292

[11] Trauzold A Siegmund D Schniewind B Sipos B Egberts J Zorenkov D. TRAIL promotes metastasis of human pancreatic ductal adenocarcinoma. Oncogene. (2006) 25:7434–9. 10.1038/sj.onc.1209719 16751802

[12] von KS Conti A Nobis M Montinaro A Hartwig T Lemke J Cancer cell-autonomous TRAIL-R signaling promotes KRAS-driven cancer progression, invasion, and metastasis. Cancer Cell. (2015) 27:561–73. 10.1016/j.ccell.2015.02.014 25843002 PMC6591140

[13] Merino D Lalaoui N Morizot A Schneider P Solary E Micheau O. Differential inhibition of TRAIL-mediated DR5-DISC formation by decoy receptors 1 and 2. Mol Cell Biol. (2006) 26:7046–55. 10.1128/MCB.00520-06 16980609 PMC1592888

[14] Neumann S Hasenauer J Pollak N Scheurich P. Dominant negative effects of tumor necrosis factor (TNF)-related apoptosis-inducing ligand (TRAIL) receptor 4 on TRAIL receptor 1 signaling by formation of heteromeric complexes. J Biol Chem. (2014) 289:16576–87. 10.1074/jbc.M114.559468 24764293 PMC4047423

[15] O'Leary L van der Sloot AM Reis CR Deegan S Ryan AE Dhami SP. Decoy receptors block TRAIL sensitivity at a supracellular level: the role of stromal cells in controlling tumour TRAIL sensitivity. Oncogene. (2016) 35:1261–70. 10.1038/onc.2015.180 26050621

[16] Chen JJ Shen HC Rivera Rosado LA Zhang Y Di X Zhang B. Mislocalization of death receptors correlates with cellular resistance to their cognate ligands in human breast cancer cells. Oncotarget. (2012) 3:833–42. 10.18632/oncotarget.542 22909995 PMC3478460

[17] Ganten TM Sykora J Koschny R Batke E Aulmann S Mansmann U. Prognostic significance of tumour necrosis factor-related apoptosis-inducing ligand (TRAIL) receptor expression in patients with breast cancer. J Mol Med. (2009) 87:995–1007. 10.1007/s00109-009-0510-z 19680616

[18] Gundlach JP Hauser C Schlegel FM Boger C Roder C Rocken C. Cytoplasmic TRAIL-R1 is a positive prognostic marker in PDAC. BMC Cancer. (2018) 18:777. 10.1186/s12885-018-4688-8 30064384 PMC6069838

[19] Haselmann V Kurz A Bertsch U Hubner S Olempska-Muller M Fritsch J. Nuclear death receptor TRAIL-R2 inhibits maturation of let-7 and promotes proliferation of pancreatic and other tumor cells. Gastroenterology. (2014) 146:278–90. 10.1053/j.gastro.2013.10.009 24120475

[20] Kojima Y Nakayama M Nishina T Nakano H Koyanagi M Takeda K. Importin beta1 protein-mediated nuclear localization of death receptor 5 (DR5) limits DR5/tumor necrosis factor (TNF)-related apoptosis-inducing ligand (TRAIL)-induced cell death of human tumor cells. J Biol Chem. (2011) 286:43383–93. 10.1074/jbc.M111.309377 22020938 PMC3234854

[21] Kriegl L Jung A Engel J Jackstadt R Gerbes AL Gallmeier E. Expression, cellular distribution, and prognostic relevance of TRAIL receptors in hepatocellular carcinoma. Clin Cancer Res. (2010) 16:5529–38. 10.1158/1078-0432.CCR-09-3403 20889918

[22] Macher-Goeppinger S Aulmann S Tagscherer KE Wagener N Haferkamp A Penzel R. Prognostic value of tumor necrosis factor-related apoptosis-inducing ligand (TRAIL) and TRAIL receptors in renal cell cancer. Clin Cancer Res. (2009) 15:650–9. 10.1158/1078-0432.CCR-08-0284 19147771

[23] Dufour F Rattier T Constantinescu AA Zischler L Morle A Ben MH. TRAIL receptor gene editing unveils TRAIL-R1 as a master player of apoptosis induced by TRAIL and ER stress. Oncotarget. (2017) 8:9974–85. 10.18632/oncotarget.14285 28039489 PMC5354785

[24] Lu M Lawrence DA Marsters S Acosta-Alvear D Kimmig P Mendez AS. Opposing unfolded-protein-response signals converge on death receptor 5 to control apoptosis. Science. (2014) 345:98–101. 10.1126/science.1254312 24994655 PMC4284148

[25] Lalaoui N Morle A Merino D Jacquemin G Iessi E Morizot A. TRAIL-R4 promotes tumor growth and resistance to apoptosis in cervical carcinoma HeLa cells through AKT. PLoS ONE. (2011) 6:e19679. 10.1371/journal.pone.0019679 21625476 PMC3098831

[26] Koksal IT Sanlioglu AD Karacay B Griffith TS Sanlioglu S. Tumor necrosis factor-related apoptosis inducing ligand-R4 decoy receptor expression is correlated with high Gleason scores, prostate-specific antigen recurrence, and decreased survival in patients with prostate carcinoma. Urol Oncol. (2008) 26:158–65. 10.1016/j.urolonc.2007.01.022 18312935

[27] Sanlioglu AD Dirice E Elpek O Korcum AF Ozdogan M Suleymanlar I. High TRAIL death receptor 4 and decoy receptor 2 expression correlates with significant cell death in pancreatic ductal adenocarcinoma patients. Pancreas. (2009) 38:154–60. 10.1097/MPA.0b013e31818db9e3 18981952

[28] Shivapurkar N Toyooka S Toyooka KO Reddy J Miyajima K Suzuki M. Aberrant methylation of trail decoy receptor genes is frequent in multiple tumor types. Int J Cancer. (2004) 109:786–92. 10.1002/ijc.20041 14999791

[29] van Noesel MM van BS Salomons GS Voute PA Pieters R Baylin SB. Tumor-specific down-regulation of the tumor necrosis factor-related apoptosis-inducing ligand decoy receptors DcR1 and DcR2 is associated with dense promoter hypermethylation. Cancer Res. (2002) 62:2157–61. 11929838

[30] Chitadze G Oberg HH Wesch D Kabelitz D. The ambiguous role of gammadelta T lymphocytes in antitumor immunity. Trends Immunol. (2017) 38:668–78. 10.1016/j.it.2017.06.004 28709825

[31] Gober HJ Kistowska M Angman L Jeno P Mori L De LG. Human T cell receptor gammadelta cells recognize endogenous mevalonate metabolites in tumor cells. J Exp Med. (2003) 197:163–8. 10.1084/jem.20021500 12538656 PMC2193814

[32] Harly C Guillaume Y Nedellec S Peigne CM Monkkonen H Monkkonen J. Key implication of CD277/butyrophilin-3 (BTN3A) in cellular stress sensing by a major human gammadelta T-cell subset. Blood. (2012) 120:2269–79. 10.1182/blood-2012-05-430470 22767497 PMC3679641

[33] Kabelitz D Marischen L Oberg HH Holtmeier W Wesch D. Epithelial defence by gamma delta T cells. Int Arch Allergy Immunol. (2005) 137:73–81. 10.1159/000085107 15832053

[34] Oberg HH Peipp M Kellner C Sebens S Krause S Petrick D. Novel bispecific antibodies increase gammadelta T-cell cytotoxicity against pancreatic cancer cells. Cancer Res. (2014) 74:1349–60. 10.1158/0008-5472.CAN-13-0675 24448235

[35] Fisher J Kramer AM Gustafsson K Anderson J. Non-V delta 2 gamma delta T lymphocytes as effectors of cancer immunotherapy. Oncoimmunology. (2015) 4:e973808. 10.4161/2162402X.2014.973808 25949890 PMC4404791

[36] Chitadze G Lettau M Bhat J Wesch D Steinle A Furst D. Shedding of endogenous MHC class I-related chain molecules A and B from different human tumor entities: heterogeneous involvement of the “a disintegrin and metalloproteases” 10 and 17. Int J Cancer. (2013) 133:1557–66. 10.1002/ijc.28174 23526433

[37] Uldrich AP Le NJ Pellicci DG Gherardin NA McPherson KG Lim RT. CD1d-lipid antigen recognition by the gammadelta TCR. Nat Immunol. (2013) 14:1137–45. 10.1038/ni.2713 24076636

[38] Xu B Pizarro JC Holmes MA McBeth C Groh V Spies T Crystal structure of a gammadelta T-cell receptor specific for the human MHC class I homolog MICA. Proc Natl Acad Sci USA. (2011) 9108:2414–9. 10.1073/pnas.1015433108 PMC3038733 21262824

[39] Wrobel P Shojaei H Schittek B Gieseler F Wollenberg B Kalthoff H. Lysis of a broad range of epithelial tumour cells by human gamma delta T cells: involvement of NKG2D ligands and T-cell receptor- versus NKG2D-dependent recognition. Scand J Immunol. (2007) 66:320–8. 10.1111/j.1365-3083.2007.01963.x 17635809

[40] Liu Z Guo B Lopez RD. Expression of intercellular adhesion molecule (ICAM)-1 or ICAM-2 is critical in determining sensitivity of pancreatic cancer cells to cytolysis by human gammadelta-T cells: implications in the design of gammadelta-T-cell-based immunotherapies for pancreatic cancer. J Gastroenterol Hepatol. (2009) 24:900–11. 10.1111/j.1440-1746.2008.05668.x 19175829

[41] Peters C Kabelitz D Wesch D. Regulatory functions of gammadelta T cells. Cell Mol Life Sci. (2018) 75:2125–35. 10.1007/s00018-018-2788-x 29520421 PMC11105251

[42] Godder KT Henslee-Downey PJ Mehta J Park BS Chiang KY Abhyankar S. Long term disease-free survival in acute leukemia patients recovering with increased gammadelta T cells after partially mismatched related donor bone marrow transplantation. Bone Marrow Transplant. (2007) 39:751–7. 10.1038/sj.bmt.1705650 17450185

[43] Helm O Mennrich R Petrick D Goebel L Freitag-Wolf S Roder C. Comparative characterization of stroma cells and ductal epithelium in chronic pancreatitis and pancreatic ductal adenocarcinoma. PLoS ONE. (2014) 9:e94357. 10.1371/journal.pone.0094357 24797069 PMC4010424

[44] Oberg HH Grage-Griebenow E Adam-Klages S Jerg E Peipp M Kellner C. Monitoring and functional characterization of the lymphocytic compartment in pancreatic ductal adenocarcinoma patients. Pancreatology. (2016) 16:1069–79. 10.1016/j.pan.2016.07.008 27424476

[45] Wilhelm M Smetak M Schaefer-Eckart K Kimmel B Birkmann J Einsele H. Successful adoptive transfer and *in vivo* expansion of haploidentical gammadelta T cells. J Transl Med. (2014) 12:45. 10.1186/1479-5876-12-45 24528541 PMC3926263

[46] Gentles AJ Newman AM Liu CL Bratman SV Feng W Kim D. The prognostic landscape of genes and infiltrating immune cells across human cancers. Nat Med. (2015) 21:938–45. 10.1038/nm.3909 26193342 PMC4852857

[47] Lo PE Dieli F Meraviglia S Tumor-infiltrating gammadelta T lymphocytes: pathogenic role, clinical significance, and differential programing in the tumor microenvironment. Front Immunol. (2014) 5:607 10.3389/fimmu.2014.00607 25505472 PMC4241840

[48] Gonnermann D Oberg HH Kellner C Peipp M Sebens S Kabelitz D. Resistance of cyclooxygenase-2 expressing pancreatic ductal adenocarcinoma cells against γ*δ* T cell cytotoxicity. Oncoim. (2014) 4:e988460. 10.4161/2162402X.2014.988460 25949900 PMC4404835

[49] Janssen O Wesselborg S Heckl-Ostreicher B Pechhold K Bender A Schondelmaier S. T cell receptor/CD3-signaling induces death by apoptosis in human T cell receptor gamma delta + T cells. J Immunol. (1991) 146:35–9. 1824593

[50] Kabelitz D Ackermann T Hinz T Davodeau F Band H Bonneville M. New monoclonal antibody (23D12) recognizing three different V gamma elements of the human gamma delta T cell receptor. 23D12+ cells comprise a major subpopulation of gamma delta T cells in postnatal thymus. J Immunol. (1994) 152:3128–36. 7511639

[51] Olofsson K Hellstrom S Hammarstrom ML. The surface epithelium of recurrent infected palatine tonsils is rich in gammadelta T cells. Clin Exp Immunol. (1998) 111:36–47. 10.1046/j.1365-2249.1998.00446.x 9472659 PMC1904845

[52] Oberg HH Kellner C Peipp M Sebens S Adam-Klages S Gramatzki M. Monitoring circulating gammadelta T cells in cancer patients to optimize gammadelta T cell-based immunotherapy. Front Immunol. (2014) 5:643. 10.3389/fimmu.2014.00643 25566256 PMC4269191

[53] Oberg HH Kellner C Gonnermann D Sebens S Bauerschlag D Gramatzki M. Tribody [(HER2)2xCD16] is more effective than trastuzumab in enhancing gammadelta T cell and natural killer cell cytotoxicity against HER2-expressing cancer cells. Front Immunol. (2018) 9:814. 10.3389/fimmu.2018.00814 29725336 PMC5916959

[54] Chen HC Dieli F Eberl M. An unconventional TRAIL to cancer therapy. Eur J Immunol. (2013) 43:3159–62. 10.1002/eji.201344105 24136367

[55] Meraviglia S Eberl M Vermijlen D Todaro M Buccheri S Cicero G. In vivo manipulation of Vgamma9Vdelta2 T cells with zoledronate and low-dose interleukin-2 for immunotherapy of advanced breast cancer patients. Clin Exp Immunol. (2010) 161:290–7. 10.1111/j.1365-2249.2010.04167.x 20491785 PMC2909411

[56] Oberg HH Kellner C Gonnermann D Peipp M Peters C Sebens S. gammadelta T cell activation by bispecific antibodies. Cell Immunol. (2015) 296:41–9. 10.1016/j.cellimm.2015.04.009 25979810

[57] Lee Y Shin JH Longmire M Wang H Kohrt HE Chang HY. CD44+ Cells in head and neck squamous cell carcinoma suppress T-cell-mediated immunity by selective constitutive and inducible expression of PD-L1. Clin Cancer Res. (2016) 22:3571–81. 10.1158/1078-0432.CCR-15-2665 26864211 PMC5623594

[58] Senbanjo LT Chellaiah MA. CD44: a multifunctional cell surface adhesion receptor is a regulator of progression and metastasis of cancer cells. Front Cell Dev Biol. (2017) 5:18. 10.3389/fcell.2017.00018 28326306 PMC5339222

[59] Shojaei H Oberg HH Juricke M Marischen L Kunz M Mundhenke C. Toll-like receptors 3 and 7 agonists enhance tumor cell lysis by human gammadelta T cells. Cancer Res. (2009) 69:8710–7. 10.1158/0008-5472.CAN-09-1602 19887600

[60] Sreeramkumar V Fresno M Cuesta N Prostaglandin E2 and T cells: friends or foes? Immunol Cell Biol. (2012) 90:579–86. 10.1038/icb.2011.75 21946663 PMC3389798

[61] Harris SG Padilla J Koumas L Ray D Phipps RP. Prostaglandins as modulators of immunity. Trends Immunol. (2002) 23:144–50. 10.1016/S1471-4906(01)02154-8 11864843

[62] Wang MT Honn KV Nie D. Cyclooxygenases, prostanoids, and tumor progression. Cancer Metastasis Rev. (2007) 26:525–34. 10.1007/s10555-007-9096-5 17763971

[63] Lo PE Pizzolato G Gulotta E Cocorullo G Gulotta G Dieli F Current advances in gammadelta T cell-based tumor immunotherapy. Front Immunol. (2017) 8:1401 10.3389/fimmu.2017.01401 29163482 PMC5663908

[64] Lo PE Di MR Pizzolato G Mocciaro F Dieli F Meraviglia S gammadelta cells and tumor microenvironment: a helpful or a dangerous liason? J Leukoc Biol. (2018) 103:485–92. 10.1002/JLB.5MR0717-275RR 29345336

[65] Paul S Lal G. Regulatory and effector functions of gamma-delta (gammadelta) T cells and their therapeutic potential in adoptive cellular therapy for cancer. Int J Cancer. (2016) 139:976–85. 10.1002/ijc.30109 27012367

[66] Silva-Santos B Serre K Norell H. gammadelta T cells in cancer. Nat Rev Immunol. (2015) 15:683–91. 10.1038/nri3904 26449179

[67] Vantourout P Hayday A. Six-of-the-best: unique contributions of gammadelta T cells to immunology. Nat Rev Immunol. (2013) 13:88–100. 10.1038/nri3384 23348415 PMC3951794

[68] Zou C Zhao P Xiao Z Han X Fu F Fu L. Gammadelta T cells in cancer immunotherapy. Oncotarget. (2017) 8:8900–9. 27823972 10.18632/oncotarget.13051 PMC5352452

[69] Schuller HM. Regulatory Role of G protein-coupled receptors in pancreatic cancer development and progression. Curr Med Chem. (2018) 25:2566–75. 10.2174/0929867324666170303121708 28260499

[70] Martinet L Jean C Dietrich G Fournie JJ Poupot R. PGE2 inhibits natural killer and gamma delta T cell cytotoxicity triggered by NKR and TCR through a cAMP-mediated PKA type I-dependent signaling. Biochem Pharmacol. (2010) 80:838–45. 10.1016/j.bcp.2010.05.002 20470757

[71] Martinet L Fleury-Cappellesso S Gadelorge M Dietrich G Bourin P Fournie JJ. A regulatory cross-talk between Vgamma9Vdelta2 T lymphocytes and mesenchymal stem cells. Eur J Immunol. (2009) 39:752–62. 10.1002/eji.200838812 19197941

[72] Dokouhaki P Schuh NW Joe B Allen CA Der SD Tsao MS. NKG2D regulates production of soluble TRAIL by *ex vivo* expanded human gammadelta T cells. Eur J Immunol. (2013) 43:3175–82. 10.1002/eji.201243150 24019170

[73] Kondo M Sakuta K Noguchi A Ariyoshi N Sato K Sato S. Zoledronate facilitates large-scale *ex vivo* expansion of functional gammadelta T cells from cancer patients for use in adoptive immunotherapy. Cytotherapy. (2008) 10:842–56. 10.1080/14653240802419328 19016372

[74] Todaro M D'Asaro M Caccamo N Iovino F Francipane MG Meraviglia S. Efficient killing of human colon cancer stem cells by gammadelta T lymphocytes. J Immunol. (2009) 182:7287–96. 10.4049/jimmunol.0804288 19454726

[75] Secchiero P Melloni E Heikinheimo M Mannisto S Di PR Iacone A. TRAIL regulates normal erythroid maturation through an ERK-dependent pathway. Blood. (2004) 103:517–22. 10.1182/blood-2003-06-2137 12969966

[76] Matsuura H Sakaue M Subbaramaiah K Kamitani H Eling TE Dannenberg AJ. Regulation of cyclooxygenase-2 by interferon gamma and transforming growth factor alpha in normal human epidermal keratinocytes and squamous carcinoma cells. Role of mitogen-activated protein kinases. J Biol Chem. (1999) 274:29138–48. 10.1074/jbc.274.41.29138 10506169

[77] Siegmund D Klose S Zhou D Baumann B Roder C Kalthoff H Role of caspases in C. Cell Signal. (2007) 19:1172–84. 10.1016/j.cellsig.2006.12.008 17291719

[78] Lemke J Noack A Adam D Tchikov V Bertsch U Roder C. TRAIL signaling is mediated by DR4 in pancreatic tumor cells despite the expression of functional DR5. J Mol Med. (2010) 88:729–40. 10.1007/s00109-010-0619-0 20354842

[79] Ding XZ Hennig R Adrian TE. Lipoxygenase and cyclooxygenase metabolism: new insights in treatment and chemoprevention of pancreatic cancer. Mol Cancer. (2003) 2:10. 10.1186/1476-4598-2-10 12575899 PMC149414

[80] Rioux N Castonguay A. The induction of cyclooxygenase-1 by a tobacco carcinogen in U937 human macrophages is correlated to the activation of NF-kappaB. Carcinogenesis. (2000) 21:1745–51. 10.1093/carcin/21.9.1745 10964107

[81] Dragovich T Burris H III Loehrer P Von Hoff DD Chow S Stratton S. Gemcitabine plus celecoxib in patients with advanced or metastatic pancreatic adenocarcinoma: results of a phase II trial. Am J Clin Oncol. (2008) 31:157–62. 10.1097/COC.0b013e31815878c9 18391600

[82] Vosooghi M Amini M. The discovery and development of cyclooxygenase-2 inhibitors as potential anticancer therapies. Expert Opin Drug Discov. (2014) 9:255–67. 10.1517/17460441.2014.883377 24483845

[83] Xu XF Xie CG Wang XP Liu J Yu YC Hu HL. Selective inhibition of cyclooxygenase-2 suppresses the growth of pancreatic cancer cells *in vitro* and *in vivo*. Tohoku J Exp Med. (2008) 215:149–57. 10.1620/tjem.215.149 18577844

[84] Sanford M. Blinatumomab: first global approval. Drugs. (2015) 75:321–7. 10.1007/s40265-015-0356-3 25637301

[85] Bargou R Leo E Zugmaier G Klinger M Goebeler M Knop S. Tumor regression in cancer patients by very low doses of a T cell-engaging antibody. Science. (2008) 321:974–7. 10.1126/science.1158545 18703743

[86] Alnaggar M Xu Y Li J He J Chen J Li M. Allogenic Vgamma9Vdelta2 T cell as new potential immunotherapy drug for solid tumor: a case study for cholangiocarcinoma. J Immunother Cancer. (2019) 7:36. 10.1186/s40425-019-0501-8 30736852 PMC6368763

[87] Fokas E O'Neill E Gordon-Weeks A Mukherjee S McKenna WG Muschel RJ. Pancreatic ductal adenocarcinoma: from genetics to biology to radiobiology to oncoimmunology and all the way back to the clinic. Biochim Biophys Acta. (2015) 1855:61–82. 10.1016/j.bbcan.2014.12.001 25489989

[88] Yagyu S Gotoh T Iehara T Miyachi M Katsumi Y Tsubai-Shimizu S. Circulating methylated-DCR2 gene in serum as an indicator of prognosis and therapeutic efficacy in patients with MYCN nonamplified neuroblastoma. Clin Cancer Res. (2008) 14:7011–9. 10.1158/1078-0432.CCR-08-1249 18980997

[89] Yang Q Kiernan CM Tian Y Salwen HR Chlenski A Brumback BA. Methylation of CASP8, DCR2, and HIN-1 in neuroblastoma is associated with poor outcome. Clin Cancer Res. (2007) 13:3191–7. 10.1158/1078-0432.CCR-06-2846 17545522

