# Supplementary material for: TRAIL-Receptor 4 Modulates γδ T Cell-Cytotoxicity Toward Cancer Cells
Source: Front Immunol. 2019 Aug 28;10:2044. doi: 10.3389/fimmu.2019.02044 (PMC6722211; doi:10.3389/fimmu.2019.02044)
Supplement: Supplementary file 1 [file Data_Sheet_1.PDF]

Supplemental Figure 1

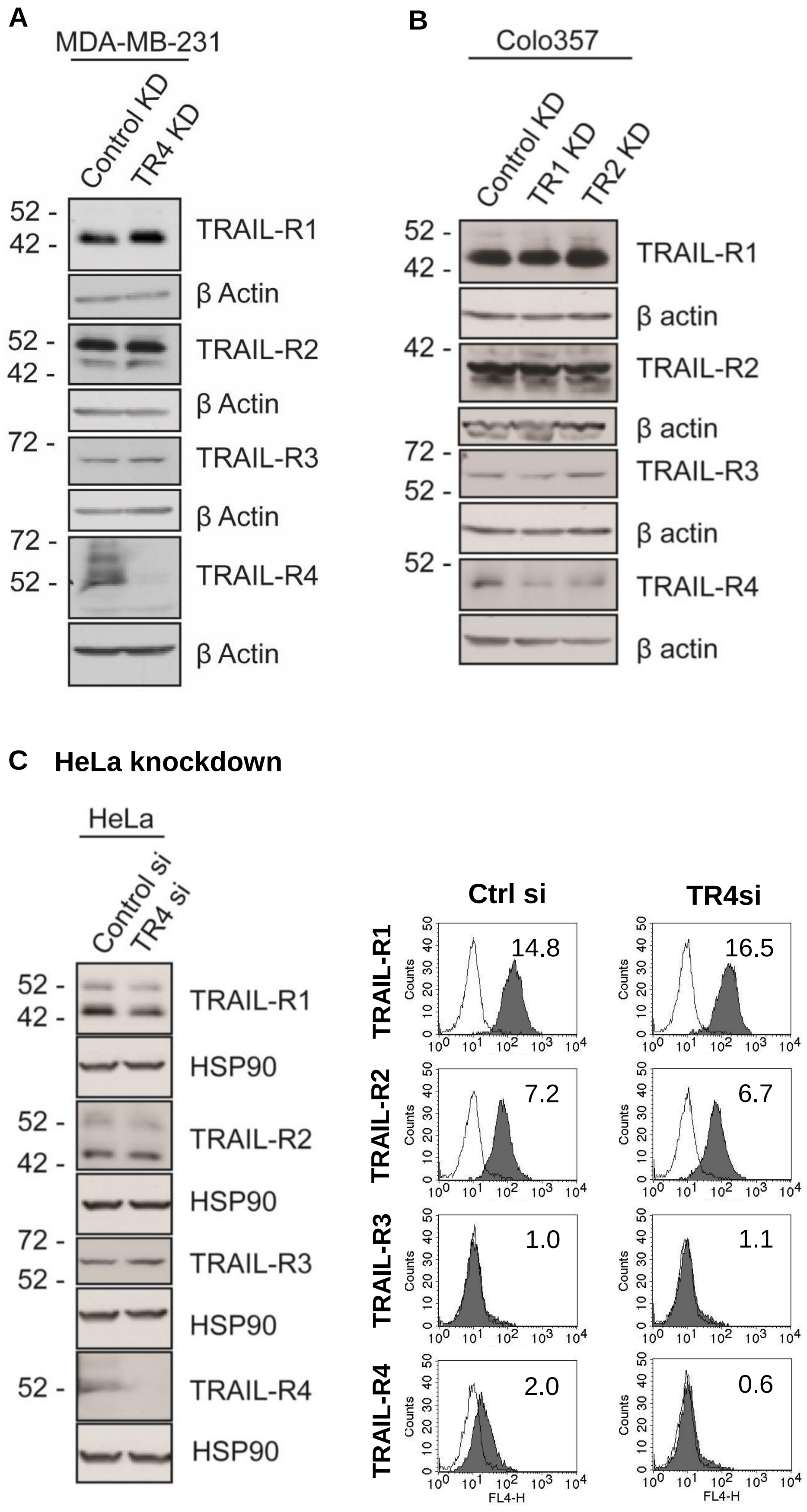

Supplemental Figure 2

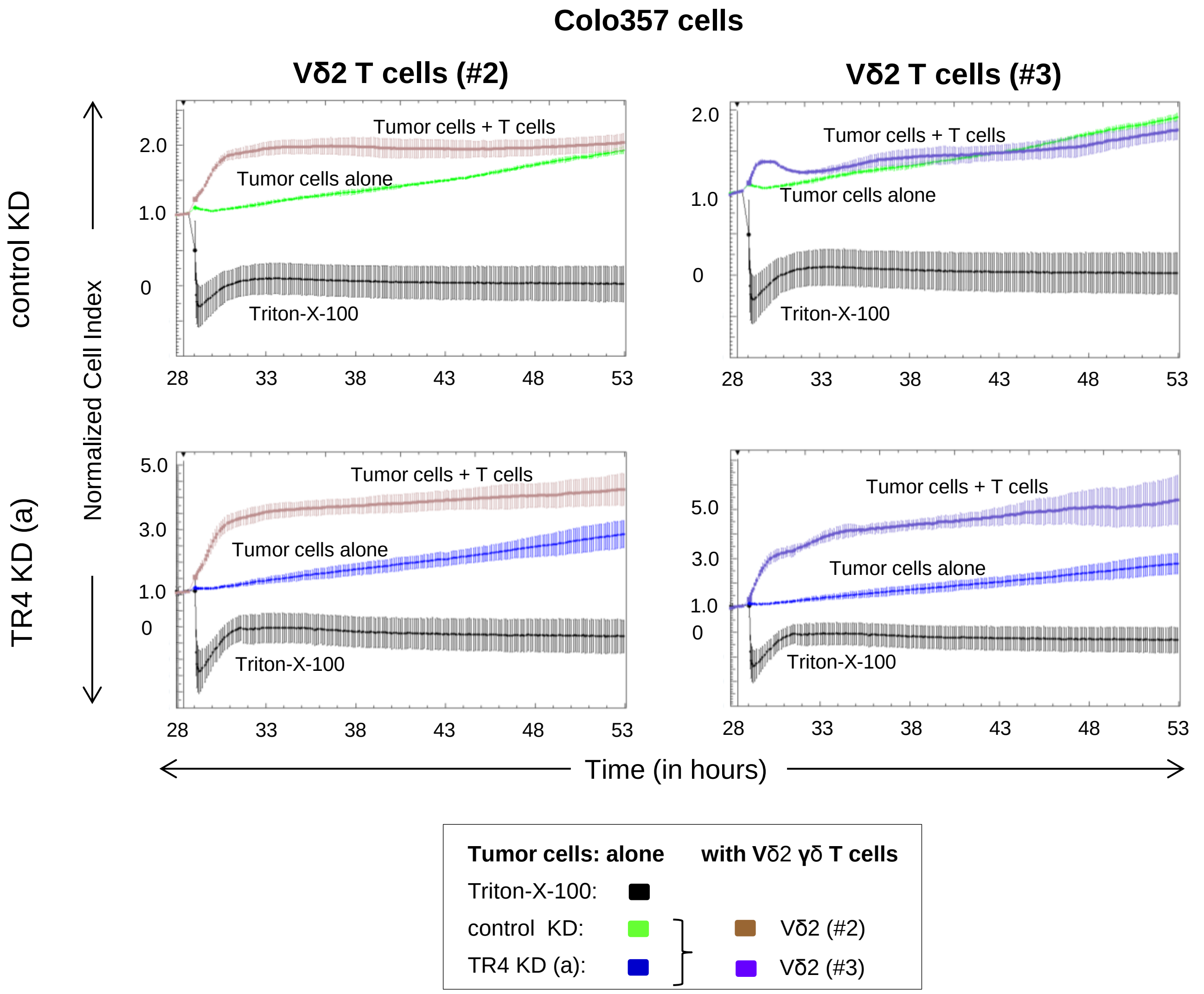

Supplemental Figure 3

HeLa TR4-knockin

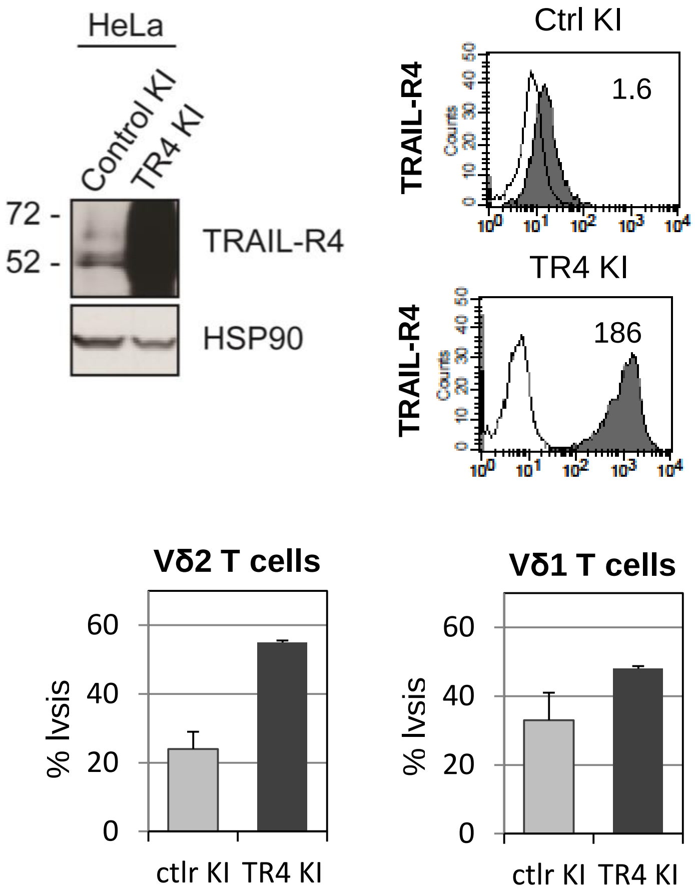

Supplemental Figure 4

Colo357 cells

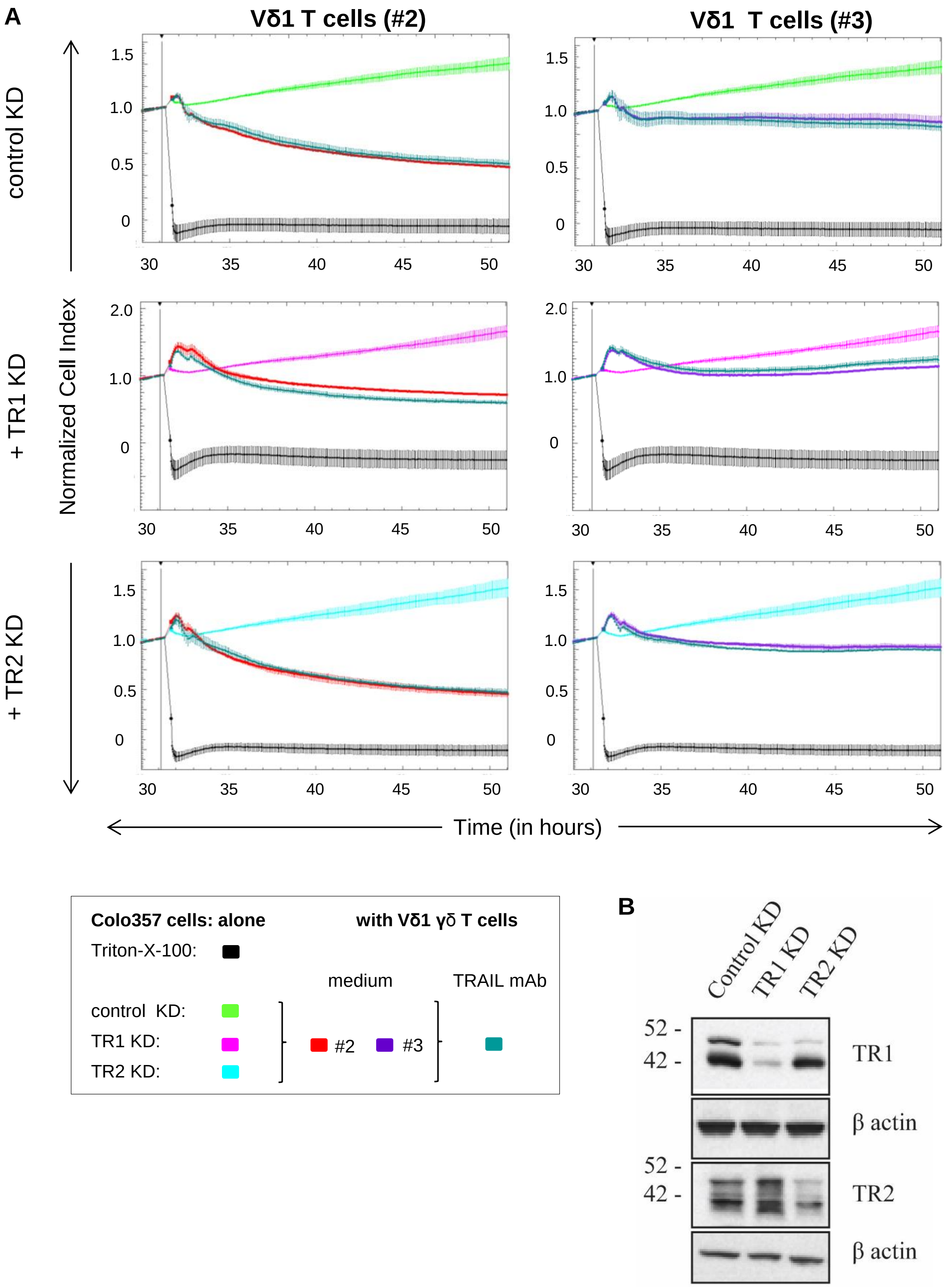

Supplemental Figure 5

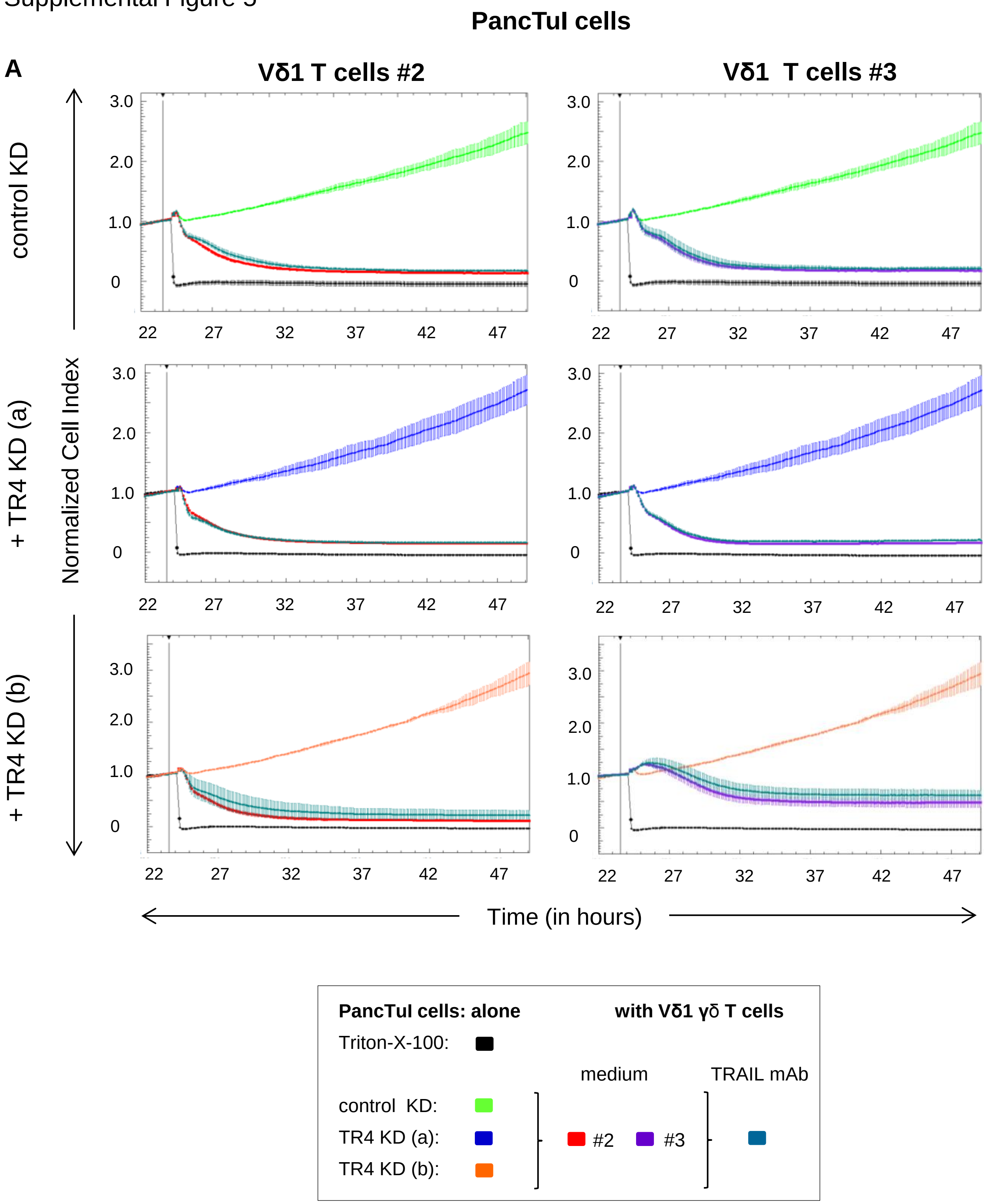

Supplemental Figure 6

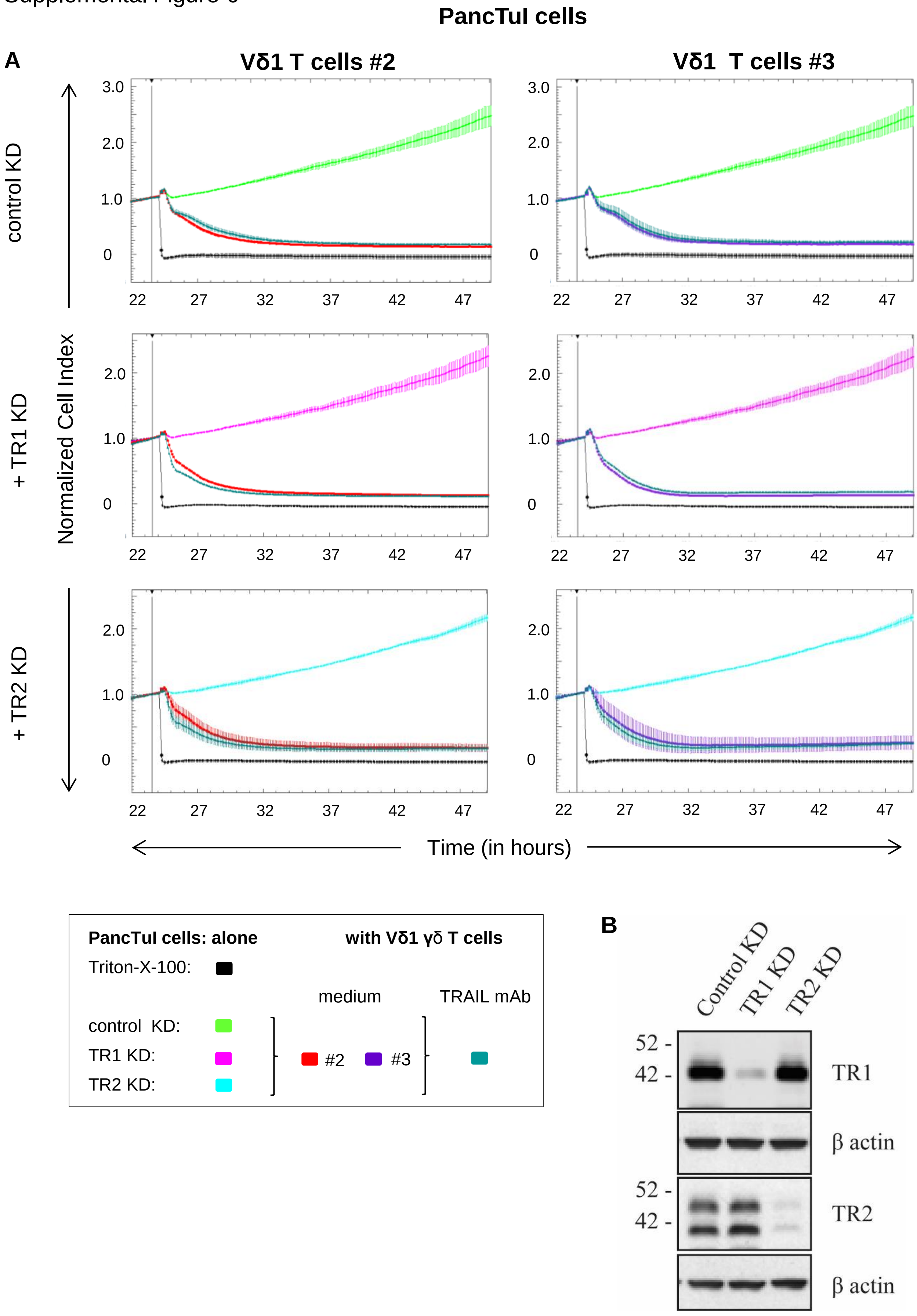

Supplemental Figure 7

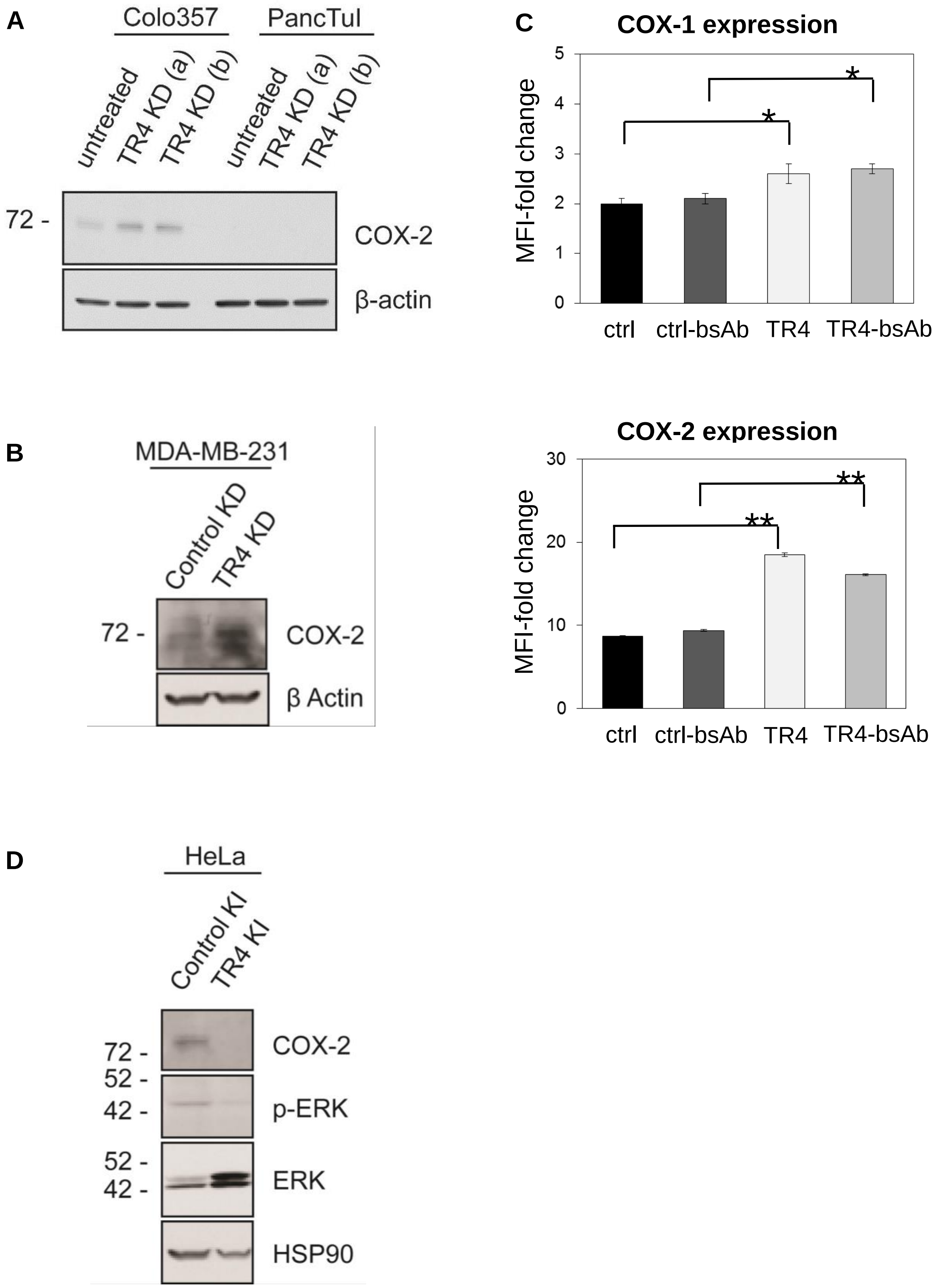

Legends to Supplemental Figures:

Suppl. Figure 1. TRAIL-receptor expression. A stable transduction of (A) MDA-MB-231 cells and (B) Colo357 cells was performed to knockdown TRAIL-R4 (TR4 KD). (A, B) Western blot analysis of the protein level of TRAIL-R1, -R2, -R3 and -R4 levels in both the control KD and TRAIL-R4 knockdown (TR4 KD) cells are shown. (C) A transient siRNA of TRAIL-R4 (TR4 si) was performed in HeLa cells. Appropriate control HeLa cells were applied. The surface expression of TRAIL-R1 to 4 was analyzed in these cells by flow cytometry. All indicated HeLa cells were stained by appropriate anti-TRAIL-R mAb (grey filled histograms) and corresponding isotype controls (thin black lines). The expression was calculated as the fold change of the median of specific antibody signal relative to the isotype control. Median of fold changes of one representative histogram out of two independent experiments is shown.

Suppl. Figure 2. Vδ2 T cell lines did not lyse Colo357 cells. Ten thousand Colo357 cells treated with control shRNA (upper panel) or with TRAIL-R4 knockdown shRNA [TR4 KD (a), lower panel] were cultured with complete medium overnight. After 28 hrs, medium (control: green line; TR4 KD: dark blue line), Triton-X-100 (black lines) or Vδ2 γδ T cell lines (E/T ratio 25:1) with 12.5 IU/mL rIL-2 of healthy donor #2 (brown lines) or pancreatic cancer patient (violet lines) were added for the indicated time points. The Cell Index (CI) was analyzed in 5 min steps over ~ 28 hrs and in 1 min steps after 28 hrs and normalization of the CI. The average of three replicates with standard deviation is presented for each line in independent experiments.

Suppl. Figure 3. TRAIL-R4 expression in HeLa knockin cells and their sensitivity to γδ T cell mediated lysis. (Upper panel) knockin TRAIL-R4 (TR4 KI) and corresponding control clone were established in HeLa cells. The surface expression of TRAIL-R4 was analyzed in these cells by using western blot and flow cytometry. All indicated HeLa cells were stained by appropriate anti-TRAIL-R4 mAb (grey filled histograms) and corresponding isotype controls (thin black lines). The expression was calculated as the fold change of the median of specific antibody signal relative to the isotype control. Median of fold changes of one representative histogram out of two independent experiments is shown. (Lower panel) Ten thousand control knockin (ctrl KI) or TRAIL-R-KI (TR4 KI) HeLa cells were cultured under the same conditions as cells described under Fig. 2A, and also cocultured with Vδ2- or Vδ1 γδ T cell lines (E/T ratio 25:1) in the presence of 12.5 IU/mL rIL-2 in

the RTCA. Percentage lysis was analyzed from RTCA data by calculating the normalized impedance of spontaneous lysis of the tumor cells in relation to the maximal lysis induced by 1% Triton-X-100. The mean of three individual samples cultured as triplicates plus standard deviation are shown 4 hrs after the addition of γδ T cells

Suppl. Figure 4. Vδ1 T cell lines lyse TRAIL-R1- and TRAIL-R2 knock down Colo357 cells. (A) After culturing 104 Colo357 cells treated with control shRNA (green lines) or with TRAIL-R1- or TRAIL-R2 knock down shRNA [TR1 KD, pink lines and TR2 KD, light blue lines] in complete medium for 32 hrs, impedance of these adherent tumor cells expressed as cell index (CI) was measured every 5 min. The CI was normalized to 1 shortly before the addition of substances as follows: Triton-X-100 (black line) or Vδ1 γδ T cell lines (E/T ratio 25:1) with 12.5 IU/mL rIL-2 of healthy donor #2 (red lines) or pancreatic cancer patient (dark purple lines) in medium or with 1 µg/mL anti-TRAIL mAb (dark green lines). CI was then measured every minute for additional 18 hrs. The average of triplicates with SD were calculated and presented for each line in independent experiments. (B) TRAIL-R1 and TRAIL-R2 levels were determined in total cell lysates of control-, TR1- and TR2 KD Colo357 cells using the indicated antibodies by western blot analysis. β actin was used as loading control.

Suppl. Figure 5. Vδ1 T cell lines lyse TRAIL-R4 knockdown PancTuI cells. (A) Impedance expressed as cell index (CI) of 104 PancTuI cells treated with control shRNA (green lines) or with TRAIL-R4 knockdown shRNA [TR4 KD (a), dark blue lines and TR4 KD (b), orange lines] was measured every 5 min for 23 hrs. The CI was normalized to 1 shortly before the addition of substances as follows: Triton-X-100 (black line) or Vδ1 γδ T cell lines (E/T ratio 25:1) with 12.5 IU/mL rIL-2 of healthy donor #2 (red lines) or pancreatic cancer patient (dark purple lines) in medium or with 1 µg/mL anti-TRAIL mAb (dark green lines). CI was then measured every minute for additional 25 hrs. The average of triplicates with SD were calculated and presented for each line in independent experiments.

Suppl. Figure 6. Vδ1 T cell lines completely lyse TRAIL-R1- and TRAIL-R2 knockdown PancTuI cells. (A) Ten thousand PancTuI cells treated with control shRNA (green lines) or with TRAIL-R1- or TRAIL-R2 knockdown shRNA [TR1 KD, pink lines and TR2 KD, light blue lines] were cultured in complete medium overnight. Impedance of these adherent tumor cells expressed as

cell index (CI) was measured every 5 min. After reaching the linear growth phase (after 23 hrs), CI was normalized to 1. Thereafter, Triton-X-100 (black line) or Vδ1 γδ T cell lines (E/T ratio 25:1) with 12.5 IU/mL rIL-2 of healthy donor #2 (red lines) or pancreatic cancer patient (dark purple lines) in medium or with 1 µg/mL anti-TRAIL mAb (dark green lines) were added to the culture. CI was then measured every minute for additional 18 hrs. The average of triplicates with SD were calculated and presented for each line in independent experiments. (B) TRAIL-R1 and TRAIL-R2 levels were determined in total cell lysates of control-, TR1- and TR2 KD PancTuI cells using the indicated antibodies by western blot analysis. β actin was used as loading control.

Suppl. Figure 7. Enhanced COX-expression of TRAIL-R4 knockdown Colo357 cells are not influenced by bispecific antibodies. Western blot analysis of (A) COX-2 expression was determined in total cell lysates of control-, TR4 (a)- and TR4 (b) KD Colo357, PancTuI, (B) MDA-MB-231 and (D) HeLa cells with anti-COX-2 mAb. In addition, the protein levels of phospho-ERK1/2 and ERK were detected in whole cell lysates. β actin or HSP90 was used as loading control. (C) Colo357 cells treated with control shRNA or with TRAIL-R4 knockdown shRNA (TR4) were cultured in medium or with 1 µg/mL bispecific antibody [HER2xCD3] for 24 hrs. Thereafter, cells were intracellularly stained with anti-Cox-1-FITC and anti-Cox-2-PE mAb mixture (AS66/AS67, BD Biosciences) and analyzed by flow cytometry. The expression is shown as fold change in median fluorescence intensity (MFI) relative to isotype control. Bars represent mean ± SD of three independent experiments ± standard deviation. Significances are shown as P Value; * = $P < 0.05$ and ** = $P < 0.01$.
